# A novel direct co-culture assay analyzed by multicolor flow cytometry reveals context- and cell type-specific immunomodulatory effects of equine mesenchymal stromal cells

**DOI:** 10.1371/journal.pone.0218949

**Published:** 2019-06-27

**Authors:** Aline Hillmann, Felicitas Paebst, Walter Brehm, Daniel Piehler, Susanna Schubert, Attila Tárnok, Janina Burk

**Affiliations:** 1 Saxon Incubator for Clinical Translation (SIKT), University of Leipzig, Leipzig, Germany; 2 Faculty of Veterinary Medicine, Equine Clinic & Hospital, University of Leipzig, Leipzig, Germany; 3 Faculty of Veterinary Medicine, Institute of Veterinary Physiology, University of Leipzig, Leipzig, Germany; 4 Horse Power Veterinary Center, Naharya, Israel; 5 Vaxxinova GmbH diagnostics, Leipzig, Germany; 6 Institute for Medical Informatics, Statistics and Epidemiology (IMISE), Faculty of Medicine, University of Leipzig, Leipzig, Germany; 7 Department of Therapy Validation, Fraunhofer Institute for Cell Therapy and Immunology, Leipzig, Germany; 8 Equine Clinic (Surgery), Justus Liebig University Giessen, Giessen, Germany; University of Central Florida, UNITED STATES

## Abstract

The immunomodulatory potential of multipotent mesenchymal stromal cells (MSC) provides a basis for current and future regenerative therapies. In this study, we established an approach that allows to address the effects of pro-inflammatory stimulation and co-culture with MSC on different specific leukocyte subpopulations. Equine peripheral blood leukocyte recovery was optimized to preserve all leukocyte subpopulations and leukocyte activation regimes were evaluated. Allogeneic labeled equine adipose-derived MSC were then subjected to direct co-culture with either non-stimulated, concanavalin A (ConA)-activated or phosphate 12-myristate 13-acetate and ionomycin (PMA/I)-activated leukocytes. Subsequently, production of the cytokines interferon-γ (IFN- γ), interleukin-1 (IL-1) and tumor necrosis factor-α (TNF-α) and presence of FoxP3 were determined in specific cell populations using multicolor flow cytometry. Prostaglandin E2 (PGE2) was measured in the supernatants. ConA-stimulation induced mild activation of leukocytes, whereas PMA/I-stimulation led to strong activation. In T cells, PMA/I promoted production of all cytokines, with no distinct suppressive effects of MSC. However, increased numbers of CD25/FoxP3-positive cells indicated that MSC supported regulatory T cell differentiation in PMA/I-activated leukocyte cultures. MSC also reduced numbers of cytokine-producing B cells and granulocytes, mostly irrespective of preceding leukocyte activation, and reversed the stimulatory effect of ConA on IFN-γ production in monocytes. Illustrating the possible suppressive mechanisms, higher numbers of MSC produced IL-10 when co-cultured with non-stimulated or ConA-activated leukocytes. This was not observed in co-culture with PMA/I-activated leukocytes. However, PGE2 concentration in the supernatant was highest in the co-culture with PMA/I-activated leukocytes, suggesting that PGE2 could still mediate modulatory effects in strongly inflammatory environment. These context- and cell type-specific modulatory effects observed give insight into the interactions between MSC and different types of immune cells and highlight the roles of IL-10 and PGE2 in MSC-mediated immunomodulation. The approach presented could provide a basis for further functional MSC characterization and the development of potency assays.

## Introduction

Multipotent mesenchymal stromal cells (MSC) have gained tremendous attention during the past decades, due to their potential as regenerative therapeutic agent for a wide range of diseases in humans and other species, such as horses. MSC are adult progenitor cells which can be isolated from virtually all vascularized tissues, while bone marrow and adipose tissue still represent the most commonly used tissue sources. Characterization of MSC, however, remains challenging and still relies on a set of inclusion and exclusion surface marker antigens, none of which alone is specific and which can vary between species and tissue sources [1, 2], as well as on plastic-adherence and trilineage differentiation into adipocytes, osteoblasts and chondrocytes in artificial *in vitro* environment [3]. This approach does not sufficiently acknowledge the functional properties of MSC. Therefore, it remains crucial to develop functional assays to complement the basic MSC characterization and gain insight into their potency, which should be based on the anticipated mechanisms of action of the cells [4, 5].

MSC are capable of homing to injured tissues and have shown the potential to engraft within lesion sites [6]. Although they are capable to differentiate into several different cell types [7], thus can potentially replace damaged cells, this mechanism is only relevant in certain contexts. Other mechanisms of action are likely to be more important and relevant for a broad range of clinical settings. These include trophic, anti-apoptotic, pro-angiogenetic, anti-fibrotic and immunomodulatory mechanisms, which rely on the secretion of cytokines, chemokines and enzymes as well as direct cell-cell contacts [8–10]. Although our understanding of these mechanisms is growing, the complex interactions between MSC, other cell types and the extracellular matrix have by far not been fully elucidated yet. Even more work is still required with regard to MSC from the equine species, as these have been investigated in fewer studies than their human or murine counterparts, while the horse is still a relevant veterinary patient and large model animal [11–15].

The immunomodulatory effects of MSC, while already being utilized clinically, such as in the treatment of graft-versus-host disease [16], have already been addressed in a broad range of experimental studies. Early studies showed that MSC inhibit T cell proliferation [17, 18] and that they can escape cytotoxic T cell lysis [19]. To date, it is known that MSC bidirectionally interact with diverse leukocyte subpopulations including B cells, natural killer cells, dendritic cells and macrophages *via* direct cell-cell-contacts as well as paracrine mediators such as interleukin-6 (IL-6), prostaglandin E2 (PGE2) or transforming growth factor β [9, 10, 20, 21]. The effects of MSC on immune cells can be suppressive as well as stimulating, depending on the context, including MSC to leukocyte ratio, pro-inflammatory priming of the MSC, or extracellular scaffold 3D environments [10, 22–24].

Given that such context-sensitive effects are evident *in vitro*, it must be anticipated that MSC can also display diverse mechanisms of action *in vivo*, which are altered by paracrine interactions as well as direct cell-cell contacts between MSC and a wide range of different cell types. However, while the importance of direct cell-cell contacts has been increasingly acknowledged in different previous study designs [25–27], the potential interplay between different leukocyte subpopulations and MSC is being largely neglected. The majority of studies have so far focused on co-culture of MSC with one specific immune cell population, typically T cells or monocytes/ macrophages [23, 24, 28, 29]. Furthermore, directly co-cultured cells or their supernatant are often analyzed as a whole, which is not conclusive regarding possibly diverse effects in the different cell types. This may be acceptable when only two different cell types are co-cultured and appropriate control monocultures are available, but aiming at better mimicry of *in vivo* conditions by co-culturing several different cell types requires more sophisticated analyses.

Multicolor flow cytometry, offering the advantage of analyzing high numbers of single cells at the same time, has recently been suggested to overcome this hurdle [30]. Flow cytometry is also suitable to analyze cytokine expression in specific cells after intracellular immunostaining, which has already been used to investigate the influence of MSC on T cells [25, 29]. Furthermore, in a possibly trend-setting approach for estimating MSC potency, flow cytometry has been used to analyze MSC-mediated suppression of monocyte activation in whole blood, as determined *via* intracellular TNF-α staining [31].

The aim of this study was to shed light on the context- and cell type-specific effects of MSC on different leukocyte subpopulations, as well as on possible underlying mechanisms, which we achieved by developing a novel direct co-culture assay with multicolor flow cytometry-based assessment.

## Materials and methods

### Study design

Peripheral blood samples from 3 healthy donor horses were used to establish the leukocyte isolation and activation protocols. In the main experiment, leukocytes were obtained from 1 healthy donor and activated using concanavalin A (ConA; mild activation) or phorbol 12-myristate 13-acetate and ionomycin (PMA/I; strong activation). They were subsequently co-cultured with adipose tissue-derived MSC from 6 further donor horses. Antibody staining and flow cytometric analysis of cell samples as well as PGE2 assay of cell culture supernatants were performed afterwards.

### Leukocyte isolation

For leukocyte recovery, peripheral blood was drawn into heparinized syringes from 3 healthy donor horses (age range: 4–15 years, male and female), as approved by the responsible authority (Landesdirektion Sachsen, TVV09/14 and N08/17), and processed immediately at room temperature. Aiming to preserve all subpopulations including granulocytes, at first, a protocol optimized for recovery of all leukocyte subpopulations was evaluated in comparison to standard density gradient centrifugation for peripheral blood mononuclear cells, using a Heraeus Multifuge X3R Centrifuge (Thermo Fisher Scientific, Darmstadt, Germany). The standard protocol included 1:3 dilution of whole blood with phosphate buffered saline (PBS) and centrifugation at 1,000 x g for 20 min without brakes using Biocoll (1.077 g/ml, Biochrom GmbH, Berlin, Germany). The optimized protocol included the following steps: A volume of 15 ml Leuko Spin Medium (1.090 g/ml, pluriSelect Life Science, Leipzig, Germany) was filled into each 50 ml LeucoSep Separating Tube (Greiner Bio One, Frickenhausen, Germany) and centrifuged at 1,000 x g for 30 s to transfer the solution beneath the separation slice. Whole blood diluted with PBS (1:2) was then carefully pipetted onto the separation slice up to a total volume of 50 ml and centrifuged at 800 x g for 20 min without brakes. After removing the supernatant above the leukocyte layer, the latter was carefully collected with a plastic pipette and washed with washing buffer (PBS with 0.5% bovine serum albumin (Sigma-Aldrich, Steinheim, Germany) and 2 mM ethylenediaminetetraacetic acid (Carl Roth GmbH & Co. KG, Karlsruhe, Germany)) at 300 x g for 10 min (brakes: level two). The supernatant was again removed, the leukocytes resuspended in washing buffer, centrifuged at 100 x g for 10 minutes (brakes: level nine) and counted. The two leukocyte recovery protocols were compared by cell counting and flow cytometric analysis focusing on the forward scatter/ side scatter distribution of the cells. The optimized protocol was applied for all further experiments.

### MSC isolation and culture

For MSC recovery, subcutaneous adipose tissue was aseptically collected from 6 different horses (age range: 2–9 years, male and female) which were systemically healthy, after they had been euthanized due to unrelated reasons such as fatal fractures. Adipose tissue was minced into small pieces of 1 mm^3^ and added to a collagenase I solution (0.8 mg/ml; Life Technologies, Karlsruhe, Germany). Subsequently, the mixture was incubated for 4 h under permanent shaking at 37°C. The nucleated cells were recovered and cultured in standard culture medium (Dulbecco’s Modified Eagle Medium (DMEM) with 1 g/l glucose, 10% fetal bovine serum (FBS), 1% penicillin-streptomycin and 0.1% gentamycin) under standard conditions (humidified atmosphere at 37°C and 5% CO_2_). Aliquots of MSC from the individual donor animals were cryopreserved in a medium consisting of 50% DMEM, 40% FBS and 10% dimethylsulfoxide at passage 1, thawed and cultured until passage 3 prior to the start of the co-culture experiments. A pooled sample containing equal numbers of MSC from all donors was additionally prepared at passage 2, cultured until passage 3 and then subjected to the same co-culture experiments as the 6 biological replicate MSC samples. This was done to evaluate the technical reproducibility of the experiments independent of inter-donor variations. Additionally, basic characteristics of MSC were confirmed at passage 3 by evaluating trilineage differentiation capacity and surface antigen expression in exemplary samples (n = 2) as described previously [25]. By adding specific differentiation media, MSC were induced to differentiate into adipogenic, osteogenic and chondrogenic lineages. Success was verified by Oil Red O staining for adipogenic differentiation, von Kossa staining for osteogenic differentiation and staining with Alcian blue for chondrogenic differentiation. CD29, CD44, CD73, CD90 and CD105 were used as inclusion markers to identify MSC and CD14, CD34, CD45, CD79α and MHCII were used as exclusion markers.

### Activation of leukocytes

Leukocytes were stimulated directly after density gradient centrifugation. First, different stimulation regimes were tested, which included incubation of leukocytes from the 3 different donors with ConA at different concentrations (1 μg/ml; 2.5 μg/ml; 5 μg/ml) and for different incubation times (1 h; 6 h), as well as incubation of leukocytes with PMA (50 ng/ml) mixed with ionomycin (1 μg/ml) (PMA/I) for 6 h (all Sigma-Aldrich). Analysis was performed by flow cytometry and focused on cell viability and IFN-γ production in the same manner as within the main experiment.

After reproducibility of leukocyte activation had been demonstrated in independent experiments, fresh leukocytes from 1 healthy donor horse (4 years, male) were obtained on one further occasion to be used throughout all co-culture experiments. Activation of leukocytes was achieved by incubating 6.4 x 10^7^ cells per 20 ml medium either with ConA (2.5 μg/ml) or with PMA/I at the concentrations given above for 6 h at standard culture conditions, as established before. A further fraction of the leukocytes was left unstimulated to be used as a control. Leukocytes were then washed and subjected to the co-culture experiments.

### Co-culture of MSC and leukocytes

**Fig 1:**
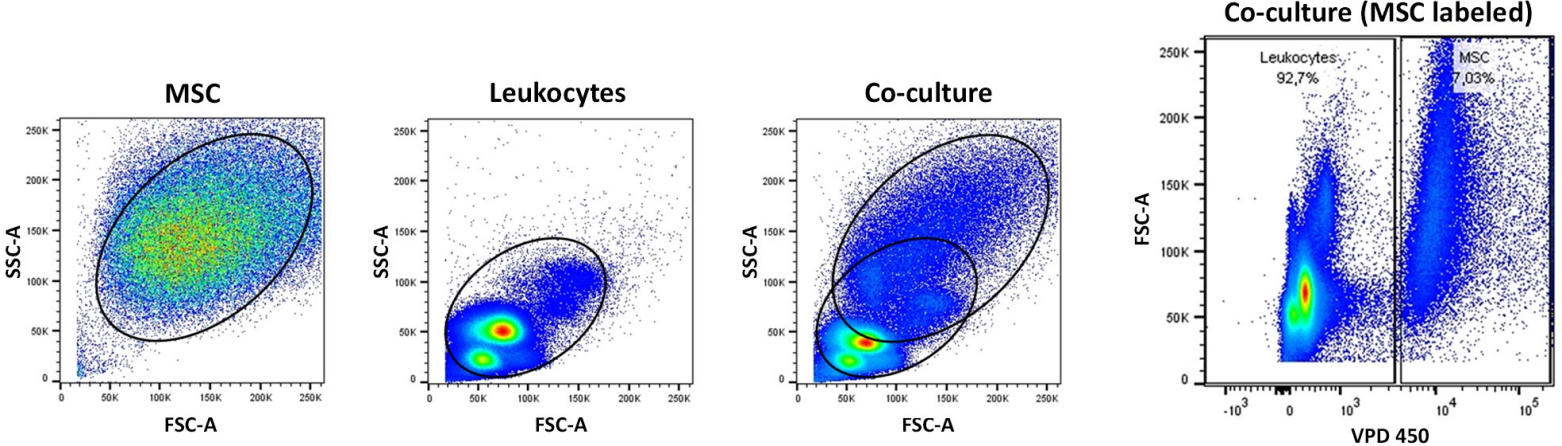
MSC discrimination based on VDP 450 labeling. The figure shows representative scatter plots of MSC and leukocytes cultured alone as well as in co-culture, analyzed by flow cytometry. In the left three plots, side scatter area (SSC-A) is plotted against forward scatter area (FSC-A), which did not enable a clear discrimination between MSC and leukocytes. Therefore, MSC were labeled with Violet Proliferation Dye 450 (VPD 450; BD) prior to co-culture. The right plot, with FSC-A being plotted against VPD 450, shows that a precise cut-off could then be placed between labeled MSC and the non-labeled leukocytes.

Directly before co-culture, all MSC were labeled with Violet Proliferation Dye 450 (VPD 450, Beckton Dickinson (BD), Heidelberg, Germany) according to the manufacturer’s instructions to enable their discrimination from co-cultured leukocytes during flow cytometry analysis (Fig 1). For co-culture, 2.4 x 10^7^ leukocytes were kept in tubes containing 20 ml standard culture medium. Labeled MSC (n = 6 biological replicates, 1 additional pooled sample) were then added to the respective leukocyte suspensions at a ratio of 1:10 [27, 29]. A suspension co-culture was chosen to better reflect the situation *in vivo* and to stimulate cell-cell contacts. Experimental groups included co-cultures of MSC and non-stimulated leukocytes, MSC and ConA-activated leukocytes, as well as MSC and PMA/I-activated leukocytes. Control groups included MSC cultures without leukocytes as well as non-stimulated, ConA-activated and PMA/I-activated leukocyte cultures without MSC. After 1 h of incubation allowing for cross-talk, to prevent rapid secretion and loss of cytokines into the culture medium, blocking of vesicle transfer from endoplasmic reticulum to Golgi apparatus was achieved by adding Brefeldin A (Brefeldin A Solution 1,000 x, Biolegend, Koblenz, Germany) according to the manufacturer’s instructions. Following another incubation time of 4 h, samples were washed with PBS, divided into equal parts for staining of the different subsets as well as staining of controls, and immediately subjected to antibody staining. The co-culture incubation time was kept short in adaptation to the life span of granulocytes.

### Antibody staining

Staining subsets were chosen to allow for analysis of the cytokines IFN-γ, IL-1, TNF-α and IL-10 in specific leukocyte subpopulations as well as MSC, and to identify FoxP3/CD25-positive regulatory T cells within the CD4-positive T cell fraction (Table 1). The gating strategies were established as described in Table 2 and the supporting information (S1–S5 Figs). All antibodies used in these multicolor flow cytometry panels had been thoroughly titrated and validated using isotype controls as well as fluorescence-minus-one (FMO) controls. All antibodies used and their dilutions are shown in the supporting information (S2 Table).

**Table 1:** Staining subsets for multicolor flow cytometry.

Subset	MSC labeling	Viability dye	Antibody staining for leukocyte discrimination		Antibody staining of cytokines/ FoxP3
1	Violet proliferation dye 450	Fixable E-Fluor 780	CD3 –FITC	CD8 –PE	IFN-γ –Alexa Fluor 647
2	Violet proliferation dye 450	Fixable E-Fluor 780	CD14 –APC	MAC–Goat anti-mouse–Alexa Fluor 700	IL-1 – Goat anti-mouse–Texas Red
3	Violet proliferation dye 450	Fixable E-Fluor 780	CD3 –FITC	CD14 –APC	TNF-α –Rabbit F(ab’)2 anti-mouse –PE
4	Violet proliferation dye 450	Fixable E-Fluor 780	CD3 –FITC	CD8 –PE	IL-10 –APC
5	Violet proliferation dye 450	Fixable E-Fluor 780	CD4 –FITC	CD25 biotinylated–Streptavidin–BV605	FoxP3 –PerCP-Cy5.5

**Table 2:** Gating for analysis of specific cell populations.

Cytokine	Cell populations analyzed	Gating
IFN-γ, IL-10	T helper cells	CD3+CD8-
	Cytotoxic T cells	CD3+CD8+
	B cells	CD3-CD8-, SSC^low^/FSC^low^
	Granulocytes	CD3-CD8-, SSC^high^/FSC^high^
	Monocytes (may include degranulated granulocytes)	CD3-CD8-, SSC^low^/FSC^high^
	MSC	VPD 450 labeling
IL-1	Lymphocytes	CD14-MAC-
	Granulocytes	CD14-MAC+
	Monocytes	CD14+MAC+
	MSC	VPD 450 labeling
TNF-α	T cells	CD3+CD14-
	B cells	CD3-CD14-, SSC^low^/FSC^low^
	Granulocytes	CD3-CD14-, SSC^high^/FSC^high^
	Monocytes	CD3-CD14+
	MSC	VPD 450 labeling

Cells were kept on ice and protected from direct light during the whole staining procedure. To enable exclusion of dead cells, viability staining was performed in all samples using Fixable E-Fluor 780 (Thermo Fisher Scientific) at a dilution of 1:1,000 for 20 min, followed by washing with PBS. Prior to surface antigen staining, cells were then incubated in a 5% heat-inactivated serum-mix fitting the hosts of the respective antibodies used (mouse and rat serum from Serotec, Kidlington, UK; rabbit and goat serum from Sigma-Aldrich; horse serum obtained from the leukocyte donor animal) for 15 min to reduce non-specific binding and then washed with PBS. All surface antigens were then stained for 15 min using the antibodies and dilutions given in the supporting information (S2 Table), if required in subsequent steps. After surface antigen staining and a subsequent washing step, cells were permeabilized with Fixation and Permeabilization Solution (BD) for 20 min. Perm/Wash Buffer (BD) was then diluted at a ratio of 1:10 with distilled water and used for two successive washing steps. Cells were then incubated with the diluted Perm/Wash Buffer for 15 min and subsequently incubated again with the serum-mix for 15 min. After another washing step with Perm/Wash Buffer, incubation with the antibodies for intracellular and intranuclear antigen staining, diluted in Perm/Wash Buffer, was performed for 15 min. This procedure was repeated as often as required for staining with all primary and secondary antibodies in the respective subset. Unstained controls and single Fixable E-Fluor 780-stained controls were prepared for each sample. After completion of the staining procedures, cells were resuspended with staining buffer (PBS with 3% fetal bovine serum and 0.1% sodium azide) and stored at 4°C until flow cytometric analysis.

### Flow cytometry

All measurements were performed on a LSR Fortessa II flow cytometer (BD) equipped with the following lasers: 488 nm (50 mW, 2 detectors), 640 nm (40 mW, 3 detectors), 561 nm (50 mW, 5 detectors), 405 nm (50 mW, 6 detectors), 355 nm (20 mW, 2 detectors) and the FACS Diva 6.1.3 software (BD). Compensation was achieved with single stained beads (UltraComp eBeads Compensation Beads and ArC Amine Reactive Compensation Bead Kit, Thermo Fisher Scientific) and calculated by the Diva software. The setup was then saved and used for all experiments. All data was analyzed by the same person using FlowJo v10.1r5 software (FlowJo, LLC, Ashland, OR, USA). During analysis, dead cells and doublets were excluded. Gating strategies focused on identification of the different cell populations and the percentages of cells in these populations positive for the respective cytokines or FoxP3. Gating boundaries were positioned based on the Live/Dead controls and remained identical during the process of analyzing.

### PGE2 assay

PGE2 concentration was determined in the cell culture supernatants using a commercially available ELISA kit (Enzo Life Sciences, Inc., Farmingdale, NY, USA). Supernatants were collected and frozen at -20°C. Before the ELISA assay was performed, thawed supernatants were filtered through a protein concentrator (Pierce Protein Concentrator PES, 10K MWCO; Thermo Fisher Scientific). PGE2 in the filtered supernatants was then analyzed according to the kit manufacturer’s instructions. Optical density was measured at 405 nm using a Synergy H1 Hybrid Multi-Mode Reader (BioTek Instruments, Bad Friedrichshall, Germany). Concentrations of PGE2 were then calculated using the standard curve and the software elisaanalysis.com.

### Statistical analysis

SPSS Statistics 23 software (IBM Deutschland GmbH, Ehningen, Germany) was used for statistical analysis. Friedman tests and post-hoc Wilcoxon signed-rank tests with correction for multiple testing were applied to analyze differences between the paired co-culture groups. Viability of MSC and leukocytes was compared using Mann-Whitney U tests. Leukocyte control cultures were not included in statistical testing as based on the study design, these data were obtained from 1 donor animal only. Correlation of results obtained with the pooled MSC with the mean and median results obtained from the biological replicate MSC samples was assessed based on Spearman-Rho. Differences were considered significant at p < 0.05.

## Results

### Characterization of MSC

The tested MSC samples showed proper differentiation into adipogenic, osteogenic and chondrogenic lineages. They expressed the inclusion markers CD29, CD44, CD90 and CD105, while CD73 was not detectable. The exclusion markers CD14, CD34, CD45, CD79α and MHCII were expressed in less than 2% of cells.

### Leukocyte recovery and activation

**Fig 2:**
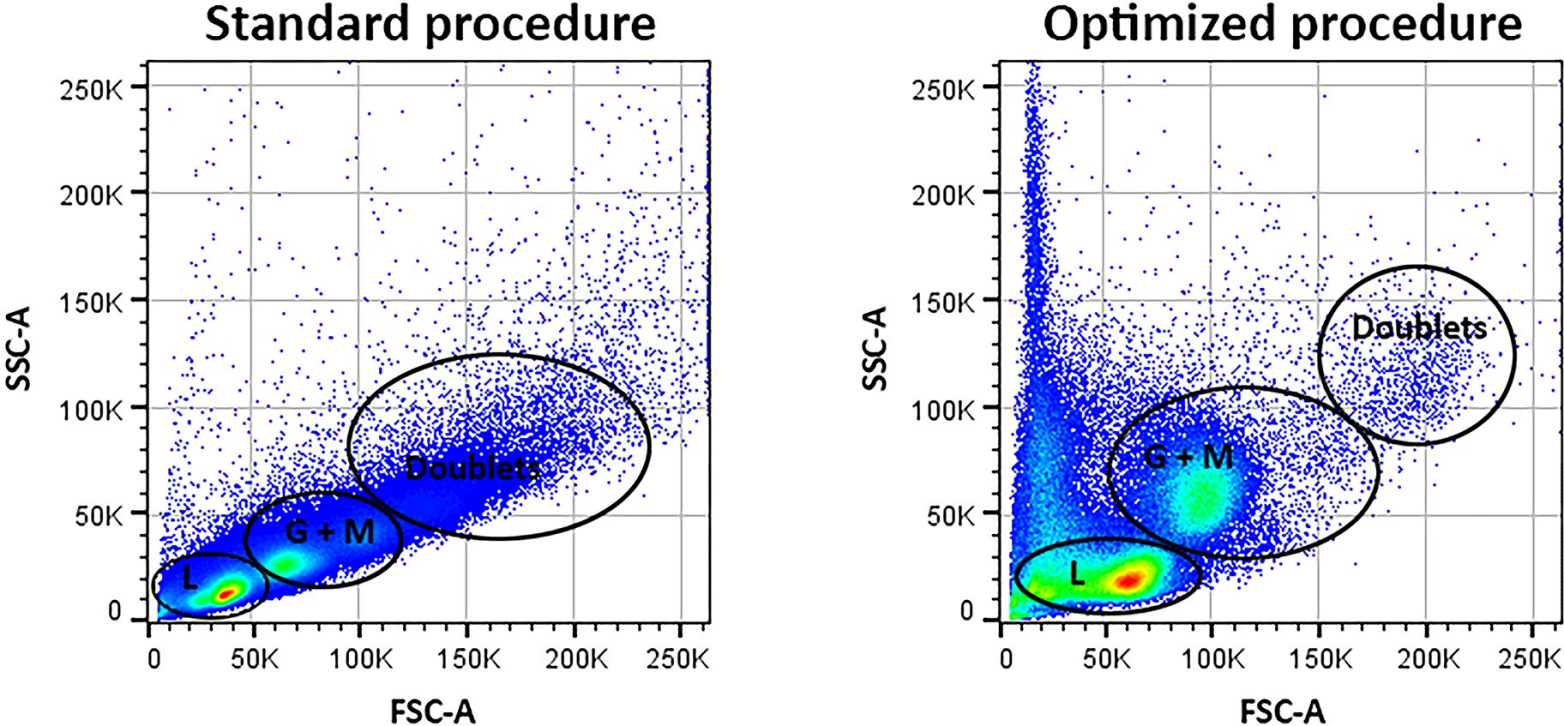
Leukocyte recovery. The scatter plots illustrate leukocyte fractions obtained following standard density gradient centrifugation (left) and the optimized procedure using Leuko Spin Medium (pluri select) (right). Cells were analyzed by flow cytometry; side scatter area (SSC-A) is plotted against forward scatter area (FSC-A). The peripheral blood leukocyte subpopulations were better preserved and less doublets were found using the optimized procedure. L: lymphocytes; G: granulocytes; M: monocytes.

Optimized density gradient centrifugation using the Leuko Spin Medium was superior to standard density gradient centrifugation in terms of preserving the granulocyte population as well as preventing their degranulation (Fig 2) and was therefore used throughout all subsequent experiments.

**Fig 3:**
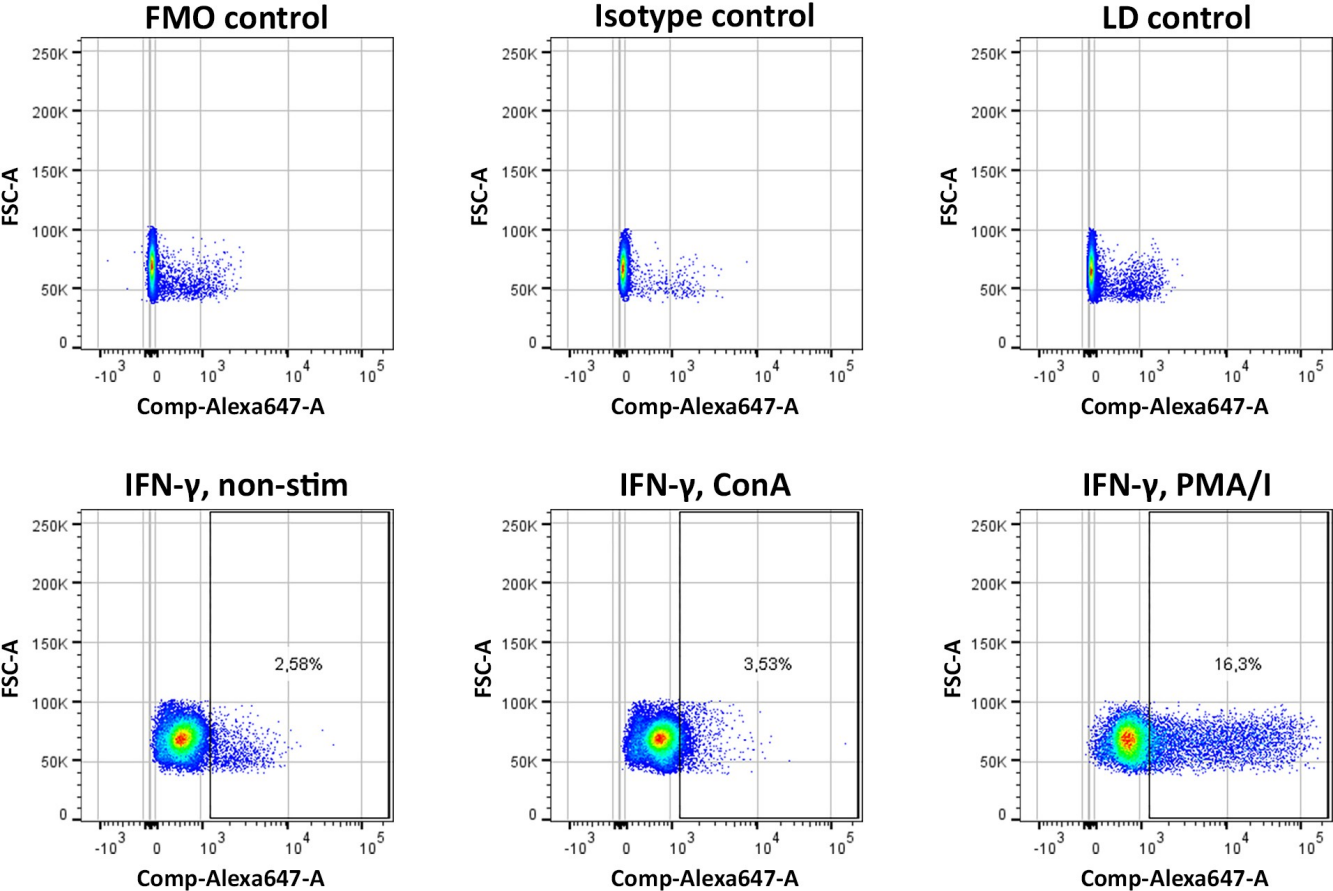
Lymphocyte activation. Representative scatter plots of lymphocytes stained for IFN-γ using a monoclonal antibody conjugated with Alexa Fluor 647 (Alexa647) after leukocytes had been left unstimulated (non-stim) or activated with ConA or PMA/I (lower row). Cells were analyzed by flow cytometry and gated based on viability staining as well as forward and side scatter. Forward scatter area (FSC-A) is plotted against Alexa647. The upper row shows all corresponding controls (FMO, isotype and viability (LD) Fixable E-Fluor 780 single stained). Data on monocyte and granulocyte populations is not shown.

From the stimulation regimes tested, ConA, used at a concentration of 2.5 μg/ml for 6 h, was chosen to reflect leukocyte activation in mild inflammation and the PMA/I stimulation regime to reflect leukocyte activation in strong inflammation in the subsequent co-culture experiments (Fig 3). ConA increased fractions of INF-γ-positive cells in the monocyte and granulocyte subpopulations (1.7 to 3.4-fold in n = 3 independent experiments), but had only minor effects on the percentage of IFN-γ-positive lymphocytes (1.1 to 1.4-fold increase in n = 3 independent experiments). Furthermore, it did not affect leukocyte viability, which remained above 95%, similar to non-stimulated leukocytes. PMA/I led to overall strong stimulation of leukocytes, including increased fractions of IFN-γ-positive lymphocytes (3.5 to 14.4-fold in n = 3 independent experiments). However, it also decreased leukocyte viability down to 70–90% and led to pronounced granulocyte degranulation, limiting their distinction based on forward and side scatter.

### Effects of activation and co-culture with MSC on leukocyte subpopulations

**Fig 4:**
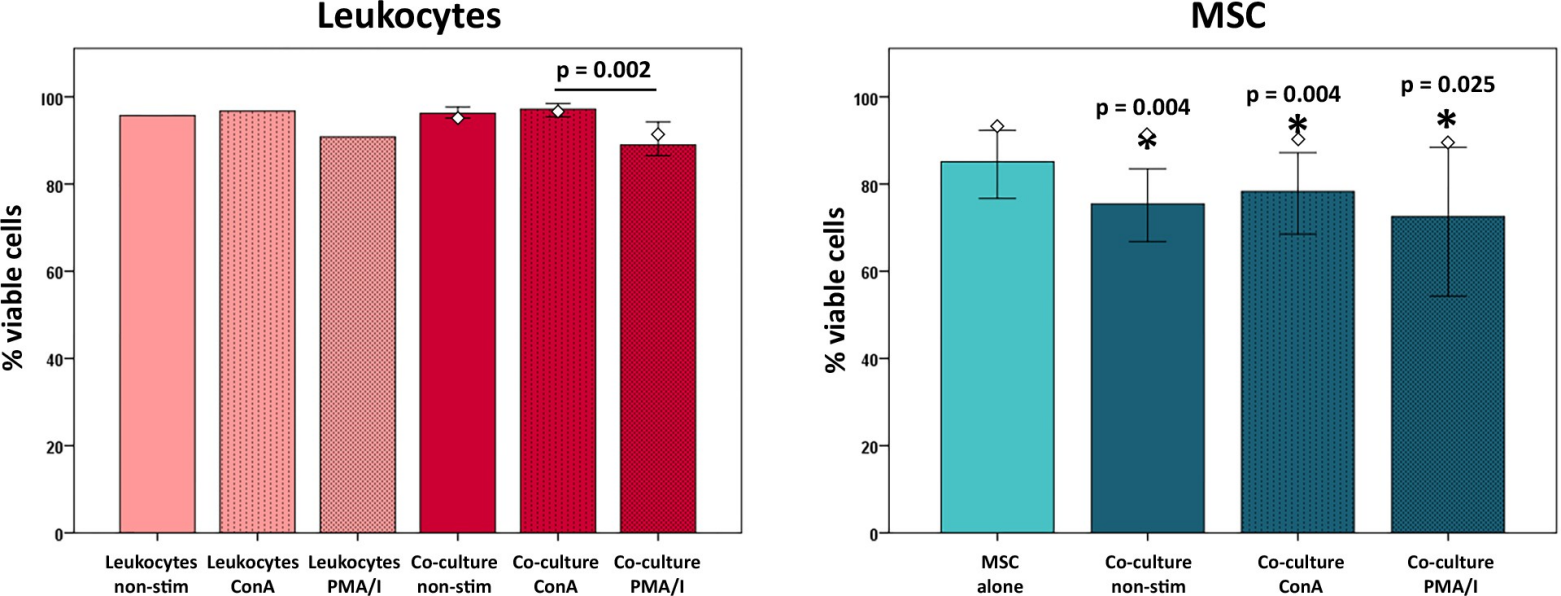
Cell viability at different culture conditions. Diagrams display the percentage of viable cells in the whole leukocyte and MSC populations, as determined by Fixable E-Fluor 780 (Thermo Fisher Scientific) staining and flow cytometry. Bars represent the median values, error bars the 95% confidence intervals. The white rhombs indicate the results obtained with the pooled MSC sample. P values in the left diagram are based on Friedman- and Wilcoxon post-hoc tests (n = 6). Stars within the right diagram indicate differences between MSC and leukocyte viability in the corresponding groups, with p values based on Mann-Whitney U-tests (n = 6).

While ConA stimulation had no major effects on overall leukocyte viability, PMA/I stimulation decreased leukocyte viability not only in the control leukocytes but also in co-culture with MSC (p < 0.05) (Fig 4).

**Fig 5:**
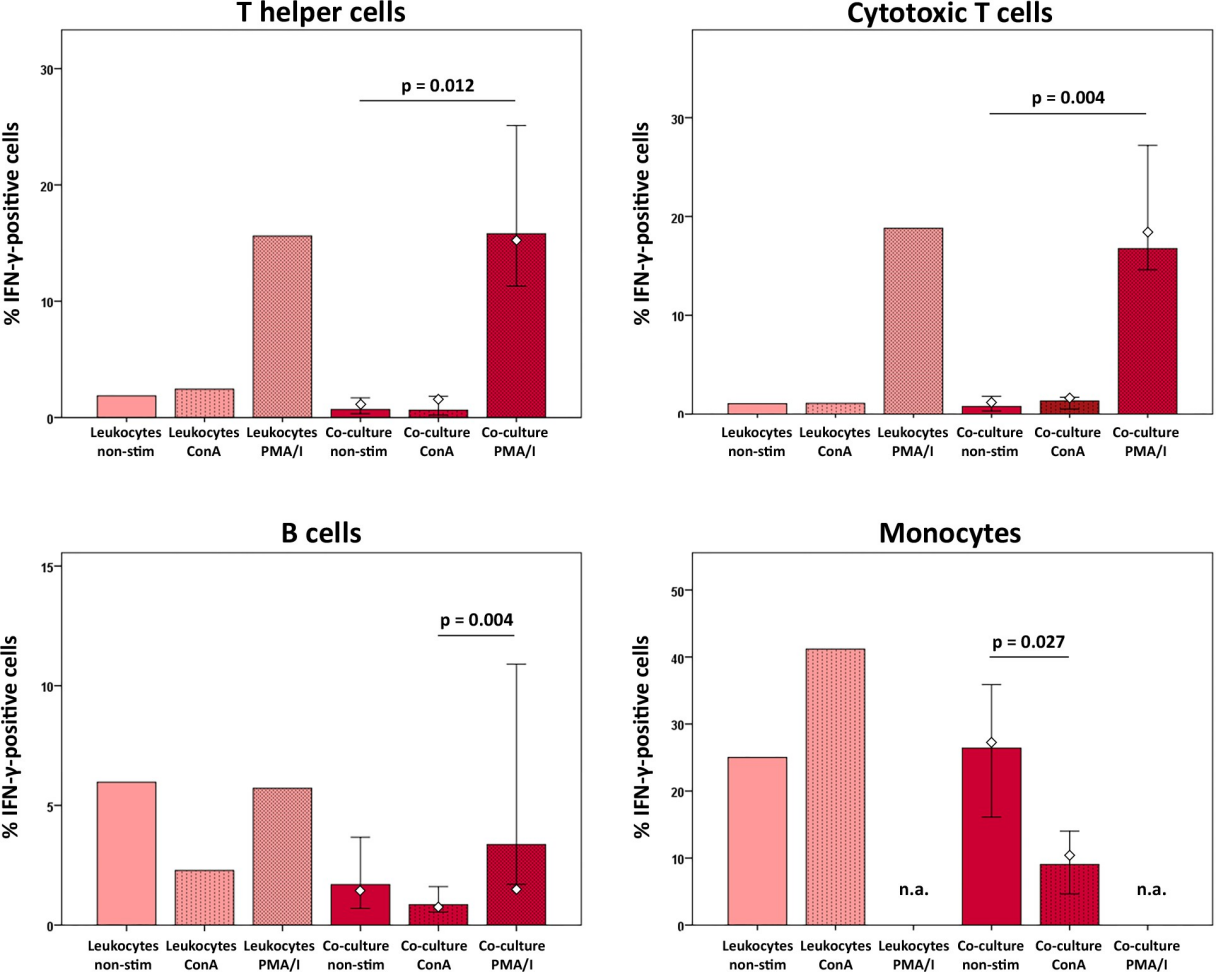
IFN-γ production in leukocyte subpopulations at different culture conditions. Data were obtained by multicolor flow cytometry following intracellular cytokine staining. Bars represent the median values, error bars the 95% confidence intervals. The white rhombs indicate the results obtained with the pooled MSC sample. P values are based on Friedman- and Wilcoxon post-hoc tests (n = 6). Groups designated as not assessed (n.a.) could not be analyzed as degranulation of granulocytes following PMA/I-stimulation had hampered monocyte-specific gating.

Proportions of IFN-γ-positive cytotoxic and helper T cells were increased by PMA/I stimulation but not by ConA stimulation, with the same trend observed in the control leukocyte groups as well as in co-cultures with MSC (p < 0.05 for the latter). Yet, percentages of IFN-γ-positive cells in the T helper and B cell fractions were lower in co-culture with MSC than in the respective counterpart leukocyte control culture groups. Moreover, in the monocyte subpopulation, the presence of MSC reversed the stimulatory effect of ConA, by decreasing the percentage of IFN-γ-positive monocytes in co-culture with ConA-stimulated leukocytes compared to the co-culture with unstimulated leukocytes (p < 0.05) (Fig 5). PMA/I-stimulated monocytes were not assessed as degranulation of granulocytes following PMA/I stimulation had hampered the monocyte-specific gating.

**Fig 6:**
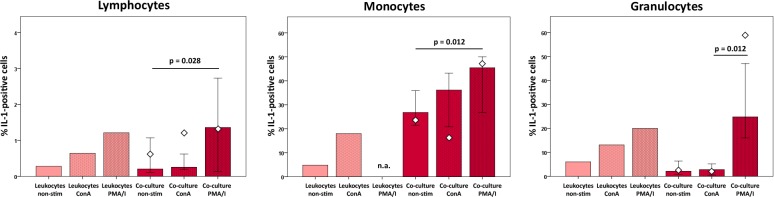
IL-1 production in leukocyte subpopulations at different culture conditions. Data were obtained by multicolor flow cytometry following intracellular cytokine staining. Bars represent the median values, error bars the 95% confidence intervals. The white rhombs indicate the results obtained with the pooled MSC sample. P values are based on Friedman- and Wilcoxon post-hoc tests (n = 6). Groups designated as not assessed (n.a.) could not be analyzed due to difficulties with gating of the monocytes due to possible CD14 surface antigen loss.

Proportions of IL-1-positive cells were higher following ConA stimulation and further increased by PMA/I stimulation in all analyzed subpopulations of control leukocyte cultures, which was also observed in the co-cultures with MSC (p < 0.05). However, in the granulocyte subpopulation, percentages of IL-1-positive cells in co-culture were lower than in the respective leukocyte control cultures, except when stimulated with PMA/I. In contrast, in the monocyte subpopulation, higher percentages of IL-1-positive cells were found in the co-cultures compared to the leukocyte control cultures (Fig 6). PMA/I-stimulated monocytes were not assessed as degranulation of granulocytes following PMA/I stimulation had hampered the monocyte-specific gating.

Proportions of TNF-α-positive cells were variable in all co-cultures with MSC and the effects of stimulation observed in leukocytes alone were neither consistently reproduced nor reversed in co-culture.

**Fig 7:**
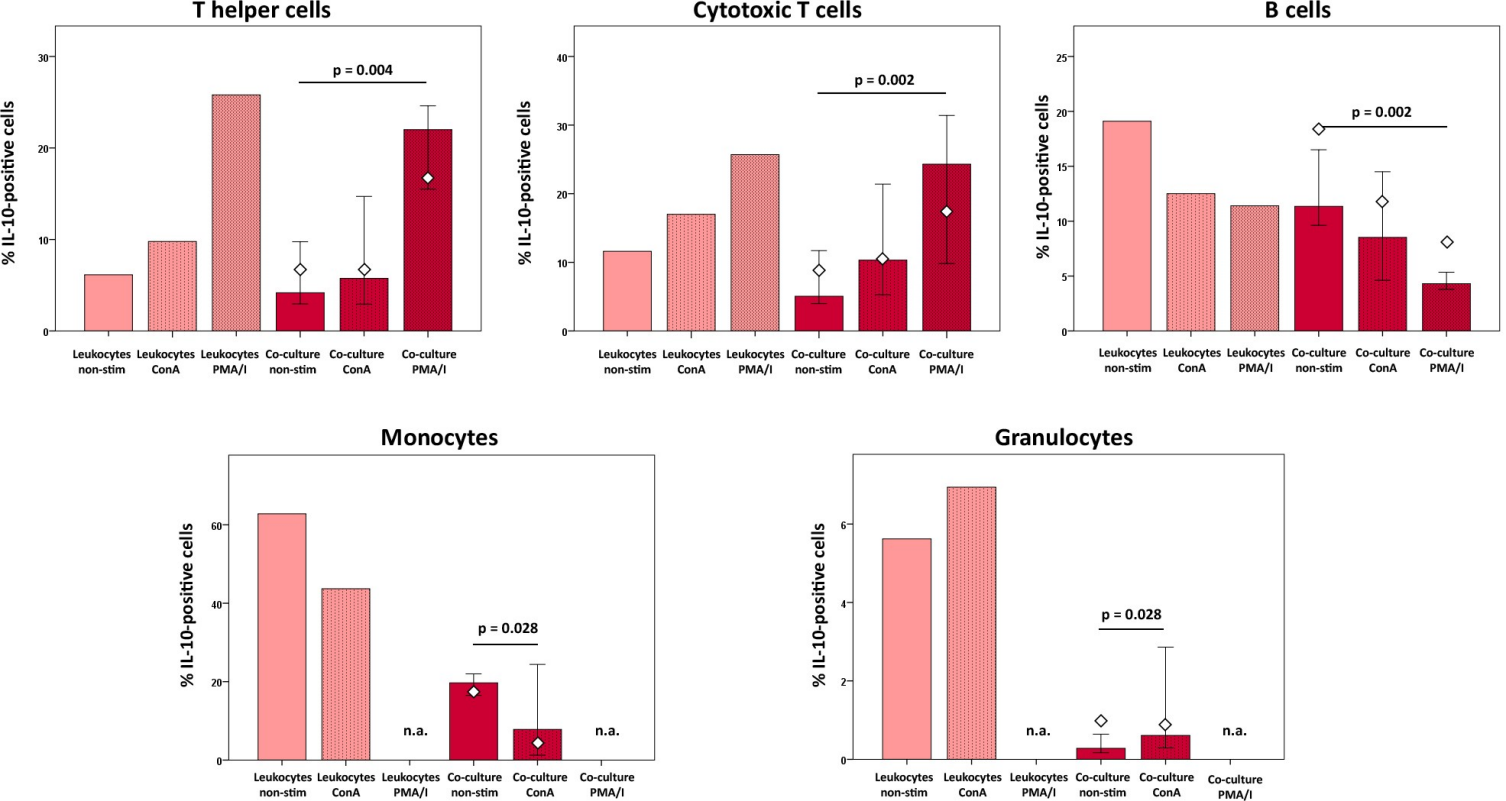
IL-10 production in leukocyte subpopulations at different culture conditions. Data were obtained by multicolor flow cytometry following intracellular cytokine staining. Bars represent the median values, error bars the 95% confidence intervals. The white rhombs indicate the results obtained with the pooled MSC sample. P values are based on Friedman- and Wilcoxon post-hoc tests (n = 6). Groups designated as not assessed (n.a.) could not be analyzed as degranulation of granulocytes following PMA/I-stimulation had hampered monocyte- and granulocyte-specific gating.

Proportions of IL-10-positive cytotoxic and helper T cells were increased upon ConA stimulation and increased further following PMA/I stimulation, which was observed in leukocyte control cultures as well as in co-culture with MSC (p < 0.05 for the co-cultures with non-stimulated *versus* PMA/I-stimulated leukocytes). An increase in IL-10-positive cells upon ConA-stimulation was also observed within the granulocyte population, in the leukocyte control culture groups as well as in co-culture with MSC (p < 0.05 for the latter). In contrast, in the B cell and monocyte subpopulations, stimulation decreased the percentage of IL-10-positive cells, as found in leukocyte control cultures as well as in co-culture (p < 0.05 for the latter, as indicated in Fig 7). Moreover, in co-culture with MSC, lower median percentages of IL-10-positive cells were present than in the counterpart leukocyte control cultures, which was observed in all leukocyte subpopulations but most distinctive in granulocytes (Fig 7). PMA/I-stimulated granulocytes and monocytes were not assessed as degranulation of granulocytes following PMA/I-stimulation had hampered distinctive gating.

**Fig 8:**
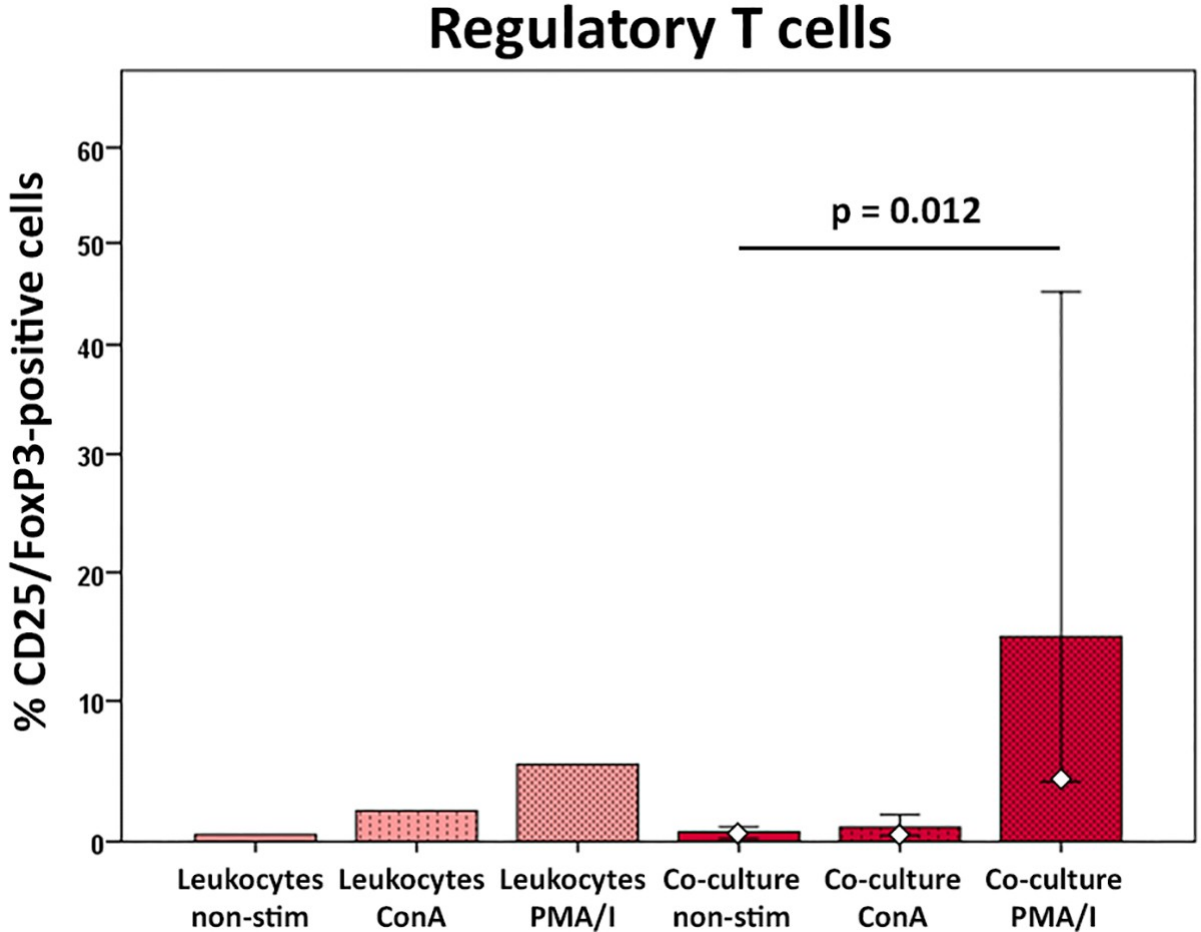
CD25/FoxP3-positive regulatory T cells at different culture conditions. Data were obtained by multicolor flow cytometry following surface antigen and intranuclear FoxP3 staining. CD25/FoxP3-positive cells are given as percentage of the CD4-positive T cell population. Bars represent the median values, error bars the 95% confidence intervals. The white rhombs indicate the results obtained with the pooled MSC sample. P values are based on Friedman- and Wilcoxon post-hoc tests (n = 6).

Percentages of CD25/FoxP3-positive regulatory cells among the CD4-positive T cells were mildly increased after ConA stimulation and strongly increased after PMA/I stimulation. Interestingly, this increase upon PMA/I stimulation was most pronounced in co-culture with MSC (p<0.05 for the co-cultures with non-stimulated *versus* PMA/I-stimulated leukocytes) (Fig 8), indicating that MSC support regulatory T cell differentiation.

Together, these data show that the effects of MSC on immune cells are not merely suppressive but rather modulatory, and that they are distinct in different cell types and inflammatory conditions. Data for all cytokines and subpopulations analyzed are presented in the supporting information (S1 Table).

### Effects of co-culture conditions on MSC

MSC viability tended to decrease in co-culture, but this was not significant. However, MSC viability in all co-culture groups was lower than leukocyte viability in the same condition (p < 0.05), which may partly be due to the MSC detachment procedure (Fig 4).

**Fig 9:**
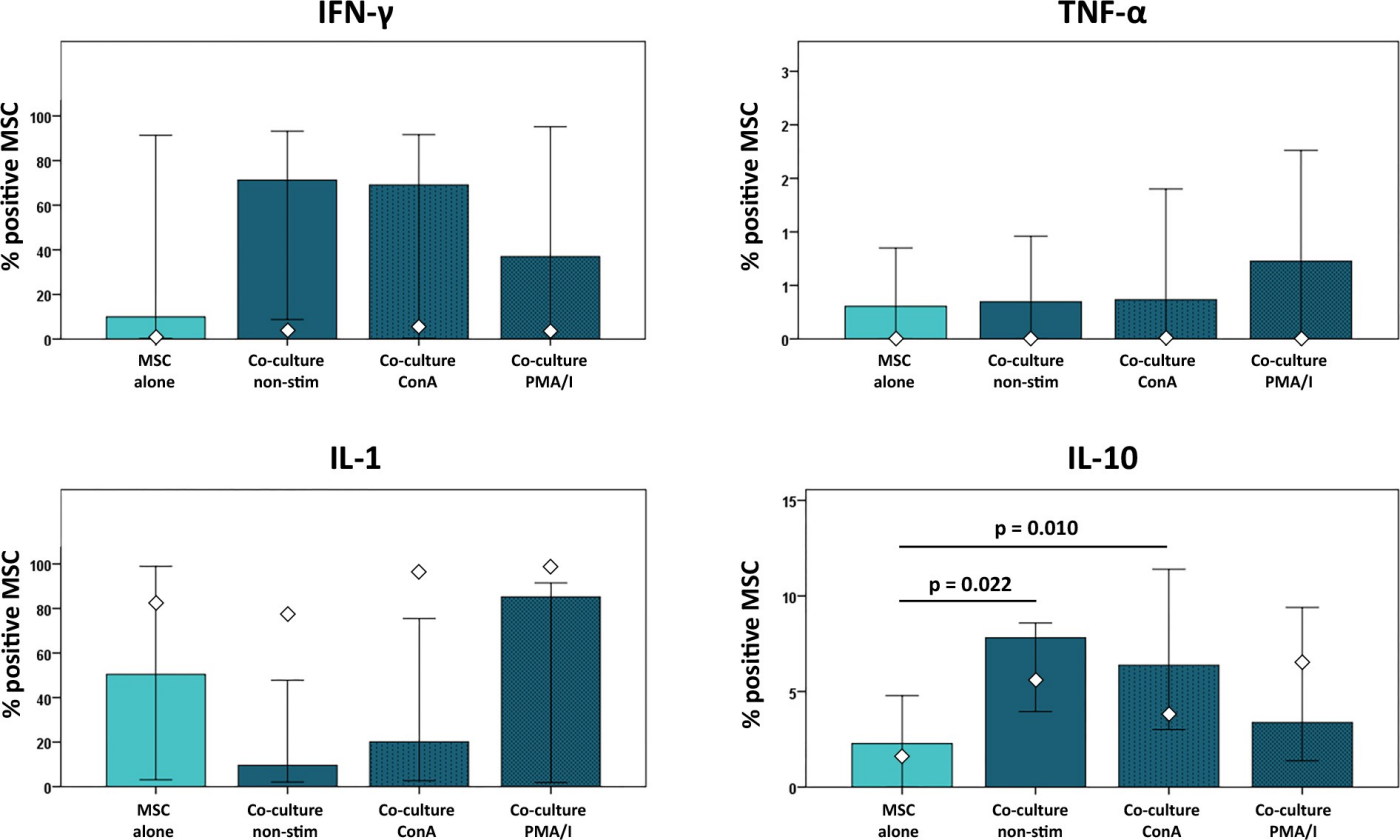
Cytokine production in MSC at different culture conditions. Data were obtained by multicolor flow cytometry following intracellular cytokine staining. Bars represent the median values, error bars the 95% confidence intervals. The white rhombs indicate the results obtained with the pooled MSC sample. P values are based on Friedman- and Wilcoxon post-hoc tests (n = 6).

Co-culture with non-stimulated and ConA-stimulated leukocytes led to increased percentages of IFN-γ- and IL-10-positive MSC compared to MSC cultured alone, whereas proportions of IL-1-positive MSC were decreased. This illustrates the induction of a modulatory MSC phenotype in the presence of leukocytes, stimulated or not. However, cytokine expression in MSC showed high variability and differences were only significant for IL-10 (p<0.05 for MSC cultured alone *versus* MSC co-cultured with non-stimulated as well as ConA-stimulated leukocytes). Co-culture with PMA/I-stimulated leukocytes did not lead to consistent effects on cytokine production in the MSC from different donors (Fig 9), suggesting that the modulatory effects of MSC in this strongly inflammatory condition were not driven by cytokine release.

### PGE2 secretion

**Fig 10:**
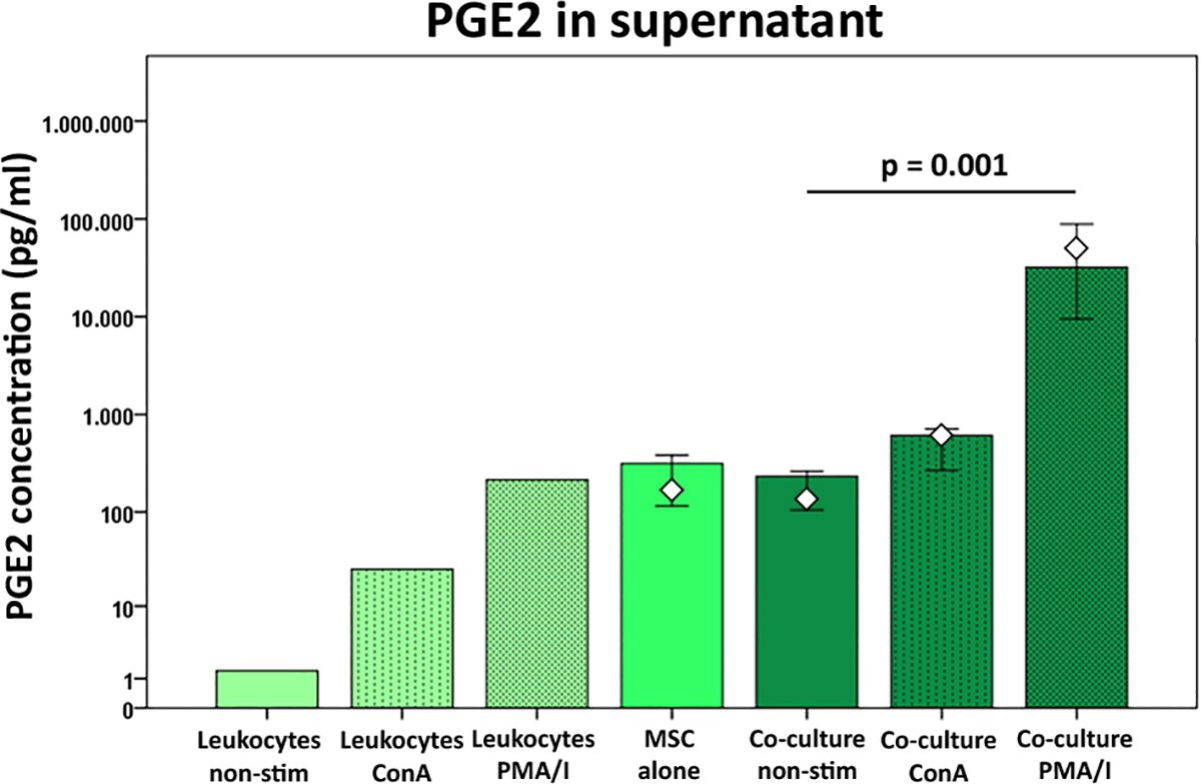
PGE2 concentrations in cell culture supernatants at different culture conditions. PGE2 was measured in the supernatants by ELISA. Bars represent the median values, error bars the 95% confidence intervals. The white rhombs indicate the results obtained with the pooled MSC sample. P values are based on Friedman- and Wilcoxon post-hoc tests (n = 6).

In the supernatants of non-stimulated as well as ConA-stimulated control leukocyte cultures, lower amounts of PGE2 were found compared to the supernatants of all MSC cultures, alone or in co-culture. Control leukocyte cultures stimulated with PMA/I produced a similar amount of PGE2 as MSC alone or co-cultures with non-stimulated leukocytes. Co-culture with activated leukocytes increased release of PGE2, which was most distinct in samples with PMA/I-stimulation (p < 0.05) (Fig 10), highlighting PGE2 as an important mediator in strong inflammation.

### Reproducibility of results with pooled MSC

Supporting reliability of data, the mean as well as median percentages of leukocyte subpopulations positive for expression of the respective cytokines, obtained based on the 6 biological MSC replicates, strongly correlated with the respective results obtained using the pooled MSC (p = 0.000). Interestingly, however, regarding cytokines measured in the MSC, although correlations were still significant (p = 0.005 for mean values; p = 0.003 for median values), data obtained with the pooled MSC did not as reliably reflect the mean or median values obtained from the 6 biological replicates. Data are presented in Figs 4–10.

## Discussion

In this study, we developed a co-culture immunomodulation assay which overcomes specific artificial conditions encountered in existing assays, in order to better reflect potential *in vivo* conditions. First of all, co-culture should allow for direct cell-cell-contacts, and secondly, co-cultured cell populations should not be restricted to MSC and one specific leukocyte subpopulation, but rather reflect all leukocyte subpopulations found in peripheral blood, as all of them contribute to immunomodulation. Furthermore, we aimed to model different inflammatory states leading to stimulation of the MSC by activated leukocytes rather than by artificially priming the MSC directly. As direct co-culture of different cell populations implicates challenges regarding reliable analysis, we established a novel multicolor flow cytometry-based approach, enabling the assessment of different cytokines in different specific cell populations within the same samples for the first time. Based on this approach, we were able to demonstrate that the immunomodulatory effects of MSC are not only context-sensitive, but also highly cell type-specific. Furthermore, our data suggest that these modulatory effects of MSC are mediated by distinct pathways, depending on the severity of inflammation, with IL-10 production being increased in mild inflammation but PGE2 release being increased in strong inflammation.

Using an optimized procedure for cell isolation from peripheral blood, we were able to include all peripheral blood leukocyte subpopulations including granulocytes, which represent a large and important fraction of leukocytes but are relatively short-lived and fragile when handled *in vitro* [32]. This acknowledged not only cell-cell interactions between MSC and leukocytes, but also between different leukocyte cell types. A procedure similarly accounting for the possible interplay between different cell types was used when MSC were cultured with diluted whole blood [31]. However, in contrast to this previous study in which analysis focused on TNF-α production in monocytes, our experimental procedure and analysis allowed to demonstrate cell-type specific immunomodulatory effects of the MSC. This is particularly valuable as firstly, data on immunomodulation by MSC which capture simultaneously occurring effects on different cell types were still lacking and secondly, the obtained results add to our understanding of MSC-granulocyte interactions, which have not yet been widely addressed so far [33].

In cytotoxic and helper T cells, the cytokine-producing fractions strongly increased upon PMA/I-stimulation, with no remarkable suppressive effect of MSC. This was unexpected as MSC have been repeatedly reported to suppress T cells, which included not only MSC-mediated suppression of T cell proliferation, but also suppressive effects on the release of cytokines such as IFN-γ, TNF-α and IL-10 [26, 27, 34, 35]. However, there was an increase of CD25/FoxP3-positive regulatory T cells after PMA/I stimulation and co-culture with MSC, demonstrating a modulatory effect of MSC on T cells, corresponding to previous findings [26, 29, 36]. As PGE2 is known to be involved in regulatory T cell differentiation, stimulating FoxP3 gene expression [37, 38], this effect may be due to the strongly increased levels of PGE2 in co-cultures of MSC and PMA/I stimulated leukocytes. Further mechanisms are likely to support the MSC-mediated FoxP3 expression in T cells, such as modification of miR-126a levels [36].

In monocytes, stimulating as well as suppressive effects of MSC and leukocyte activation were evident. On the one hand, co-culture with MSC combined with preceding stimulation of leukocytes increased percentages of cells positive for the pro-inflammatory cytokine IL-1 and decreased percentages of cells positive for the anti-inflammatory IL-10. On the other hand, it decreased the percentage of IFN-γ-positive monocytes, which could be considered a suppressive effect. Interestingly, however, percentages of MSC producing the respective cytokines showed the opposite trend in the different culture conditions. This effect would not have been revealed by supernatant analyses and suggests a strong interplay between MSC and monocytes. In this line, a recent study highlighted monocytes as key players in mediating the immunomodulatory effects of MSC, at which phagocytosis followed by monocyte phenotype changes played a major role [39]. Correspondingly, MSC have repeatedly been reported to be involved in macrophage M1/ M2 phenotype switching, at which again, PGE2 played a crucial role [28, 40]. Potentially, the pro-inflammatory and suppressive effects of MSC on monocytes observed in the current study correspond to distinct monocyte subpopulations, yet this remains to be elucidated in future studies.

In granulocytes as well as B cells, MSC appeared to suppress production of pro-inflammatory but also anti-inflammatory cytokines. However, the pattern in which these cells reacted upon stimulation was largely unchanged in co-culture, thus no impact of the different inflammatory states on the effects of MSC on these cell types was evident. In contrast to the well-characterized interactions between MSC and T cells or monocytes/ macrophages, fewer data on MSC-granulocyte and MSC-B cell interactions are available so far [22, 33, 41]. Our observation might correspond to a recent study in which MSC were shown to suppress neutrophil-mediated tissue damage [33], strongly encouraging further investigation. However, data on B cells are partly conflicting, as previous studies demonstrated a context-dependent MSC-mediated increase in IL-10 production by B cells [22], which stands in contrast to the current data.

Due to the context-sensitivity of cell-cell interactions, experimental settings chosen strongly impact on the results of studies on immunomodulation by MSC, thus apparently contradictory results are likely due to different experimental conditions. In addition to the cell populations co-cultured and the co-culture procedure itself (indirect vs direct co-culture), differences in study designs include MSC to leukocyte ratios, co-culture incubation times as well as regimes for leukocyte activation or MSC priming.

The MSC to leukocyte ratio of 1:10 in the current study was chosen based on previous reports, which had demonstrated that this ratio is more effective in terms of T cell suppression than lower ratios; furthermore, a ratio of 1:10 has also been used for MSC-macrophage-co-cultures [27, 29, 40]. However, in other studies, a ratio of 2:15 was considered most suitable for co-culture of MSC and peripheral blood mononuclear cells with respect to suppression of IgG production [25], and a ratio of 1:5 has been used to investigate interactions of MSC and B cells [22]. However, unfortunately, direct comparison of MSC to leukocyte ratio is impossible due to the different cell types co-cultured in different studies.

Co-culture incubation times in other studies were often longer than in the current experiments. For example, suppressive effects of MSC on T cell proliferation were reported after a co-culture period of 4 days [27], and suppressive effects of MSC on Th1 differentiation and INF-γ production were observed 6 days after differentiation was initiated [29]. In the current study, incubation times of leukocytes and co-cultures were kept short to allow for analysis of the short-lived granulocytes, which may have hampered detection of some effects of MSC on T cells. However, similar as in the current study, co-cultures of MSC and macrophages or MSC and peripheral blood cells were incubated for a period of 6 h to assess effects of MSC on macrophages or monocytes, respectively [23, 31]. Incubation times following leukocyte activation or in co-culture are also of particular interest regarding the detection of intracellular cytokines, as different cytokines follow diverse kinetics [31], making it difficult to choose the optimal time point for analyzing different cytokines at the same time. This may have obscured potential effects of MSC on TNF-α production, although the protocol described allowed to detect immune cell responses to inflammatory stimulation for all cytokines investigated.

Inflammatory priming of MSC has repeatedly been demonstrated to increase their immunosuppressive effects [23, 27, 42–44]. However, direct priming of MSC *in vitro* does not correspond to current clinical scenarios and relies on artificial stimulation, the long-term effects of which are not yet known. Therefore, we used non-primed MSC but allowed for cross-talk with the leukocytes in co-culture prior to blocking cytokine secretion. While direct priming might have led to even more pronounced effects, we still observed context-sensitivity of MSC effects, such as that suppression of IFN-γ production in monocytes was only evident in co-cultures with activated leukocytes. With strong clinical relevance, this illustrates that MSC were primed by the activated leukocytes, supporting that MSC mechanisms of action *in vivo* depend on the inflammatory state of the disease.

Indeed, distinct modulatory mechanisms were induced in the different inflammatory conditions. While more MSC produced IL-10 in mild inflammatory conditions, PGE2 release but not IL-10 production by MSC was increased in strong inflammatory conditions. Both mediators display anti-inflammatory properties and particularly PGE2 has repeatedly been associated with immunosuppressive effects of MSC [45]. The increased PGE2 release could be induced by the increased IFN-γ and IL-1 production by several cell types in the co-cultures with PMA/I activated leukocytes, as IFN-γ induces cyclooxygenase-2 [46] and PGE2 is known to be part of the IL-1 regulatory feedback loop [47–49]. PGE2 is also implicated in the regulation of IL-10, with stimulatory effects observed in most studies [50–52]. Although this was not evident in the current study, most likely due to the relatively short co-culture period, regulatory T cell differentiation was observed, which can give rise to IL-10-producing cells [53]. Moreover, PGE2 plays a key role in modulating macrophage plasticity [54] and supports the maintenance and migration of MSC [55]. Correspondingly, levels of PGE2 release correlated with therapeutic efficacy of MSC [56], thus the increased PGE2 release in strong inflammation indicates the potency of the cells.

The experiments within this study were carried out with equine cells, as they are not only readily available in high numbers and a valuable model for human applications, but also of direct relevance for regenerative therapies in veterinary medicine. Providing a basis for the current experiments, compared to MSC from other large animal species, immunomodulation by equine MSC is already relatively well-characterized [23, 27, 57–59]. Furthermore, we have previously demonstrated a great extent of similarity between equine MSC and their human counterparts [1]. Finally, the equine blood count with its high proportion of granulocytes reflects the human blood count very well [60, 61]. However, while we believe that the equine *in vitro* model is very valuable due to these reasons, it needs to be acknowledged that working with equine material also leads to limitations, such as that availability of anti-equine monoclonal antibodies confines the possible multicolor flow cytometry staining subsets. For example, illustrating this challenge, recent data document that staining with the anti-CD8 antibody used in this work (clone CVS21) also identifies some CD3-positive cells [62]. This had not been described when the work reported here was performed, but may have some, although presumably minor, impact on the current results.

Using cells from a large animal species also allowed to investigate true biological replicates, reflecting potential variability between MSC from different donors, which would also occur in human MSC [1, 31]. This inter-donor variability can hamper reproducibility of experiments with different biological replicates. A commonly used procedure to overcome this is to pool the MSC from different donors and to perform independent experiments with this pool of MSC. However, while demonstrating technical reproducibility of experiments, this neglects that inter-donor variability is also to be expected in clinical settings. Therefore, we chose to use both biological replicates as well as a pool of MSC from all donors for all experiments, demonstrating that while inter-donor variability was evident, technical reproducibility was high.

## Conclusions

The current study illustrates that the context-dependent immunomodulatory effects of MSC are mediated by different mechanisms, with either IL-10 or PGE2 as key mediator, depending on the severity of inflammation. The remarkable cell type-specificity of effects observed underlines the importance of investigating immunomodulation in settings that best possibly mimic and recapitulate the complexity of cell-cell interactions occurring *in vivo*. The approach presented provides a promising basis for the development of future functional MSC characterization assays and to gain further insight into specific cell-cell interactions occurring under the influence of MSC.

## Supporting information

S1 Table Cytokine production and CD25/FoxP3 expression in leukocyte subpopulations. Data are presented as median (minimum–maximum) percentage of cells positive for the respective cytokine or CD25/FoxP3. When designated as not assessed (n.a.), reliable gating and analysis of the respective cell population was not possible due to degranulation or surface antigen loss.
(PDF)

S2 Table Primary antibodies, isotype controls and secondary antibodies. (PDF)

S1 Fig Gating strategies IFN-γ. The figure shows systematic gating strategies based on FMO, isotype and Live/Dead controls for samples stained with CD3, CD8 and IFN-γ. A co-cultured sample of MSC and ConA-activated leukocytes was used to create the figure.
(PDF)

S2 Fig Gating strategies IL-10. The figure shows systematic gating strategies based on FMO, isotype and Live/Dead controls for samples stained with CD3, CD8 and IL-10. A co-cultured sample of MSC and ConA-activated leukocytes was used to create the figure.
(PDF)

S3 Fig Gating strategies TNF-α. The figure shows systematic gating strategies based on FMO, isotype and Live/Dead controls for samples stained with CD3, CD14 and TNF-α. A co-cultured sample of MSC and ConA-activated leukocytes was used to create the figure.
(PDF)

S4 Fig Gating strategies IL-1. The figure shows systematic gating strategies based on FMO, isotype and Live/Dead controls for samples stained with CD14, MAC and IL-1. A co-cultured sample of MSC and ConA-activated leukocytes was used to create the figure.
(PDF)

S5 Fig Gating strategies FoxP3. The figure shows systematic gating strategies based on FMO, isotype and Live/Dead controls for samples stained with CD4, CD25 and FoxP3. A co-cultured sample of MSC and ConA-activated leukocytes was used to create the figure.
(PDF)
