# Supplementary material for: A novel direct co-culture assay analyzed by multicolor flow cytometry reveals context- and cell type-specific immunomodulatory effects of equine mesenchymal stromal cells
Source: PLoS One. 2019 Jun 27;14(6):e0218949. doi: 10.1371/journal.pone.0218949 (PMC6597077; doi:10.1371/journal.pone.0218949)
Supplement: S1 Table — Data are presented as median (minimum–maximum) percentage of cells positive for the respective cytokine or CD25/FoxP3. When designated as not assessed (n.a.), reliable gating and analysis of the respective cell population was not possible due to degranulation or surface antigen loss. (PDF) [file pone.0218949.s001.pdf]

S1 Table:

Cytokine production and CD25/FoxP3 expression in leukocyte subpopulations

Data are presented as median (minimum – maximum) percentage of cells positive for the respective cytokine or CD25/FoxP3. When designated as not assessed (n.a.), reliable gating and analysis of the respective cell population was not possible due to degranulation or surface antigen loss.

		**Leukocytes alone, non-stim**	**Leukocytes alone, ConA**	**Leukocytes alone, PMA/I**	**Co-culture, non-stim**	**Co-culture, ConA**	**Co-culture, PMA/I**
IFN-γ	T helper cells	1.9	2.4	15.6	0.7 (0.3-1.7)	0.6 (0.2-1.8)	15.8 (11.3-25.1)
	Cytotoxic T cells	1.1	1.1	18.8	0.8 (0.3-1.8)	1.3 (0.5-1.7)	16.8 (14.6-27.2)
	B cells	6.0	2.2	5.7	1.7 (0.7-3.7)	0.9 (0.5-1.6)	3.4 (1.7-10.9)
	Granulocytes	4.1	7.3	n.a.	1.3 (0.6-18.7)	2.2 (0.5-16.5)	n.a.
	Monocytes	25.0	41.2	n.a.	26.4 (16.1-35.9)	9.1 (4.6-14.0)	n.a.

TNF-α	T cells	0.0	0.1	0.2	0.1 (0.0-0.4)	0.1 (0.1-1.3)	0.1 (0.0-0.1)
	B cells	0.4	0.7	n.a.	0.4 (0.2-0.5)	0.3 (0.2-0.6)	0.3 (0.2-0.4)
	Granulocytes	0.4	0.6	1.9	0.4 (0.2-1.2)	0.4 (0.2-1.6)	n.a.
	Monocytes	0.5	1.8	n.a.	0.2 (0.1-3.0)	0.2 (0.0-2.5)	1.1 (0.0-5.1)
IL-1	Lymphocytes	0.3	0.6	1.2	0.2 (0.1-1.1)	0.3 (0.2-0.6)	1.4 (0.1-2.7)
	Granulocytes	6.0	13.1	20.0	2.1 (0.8-6.4)	2.8 (0.5-5.2)	24.8 (16.8-47.0)
	Monocytes	4.8	18.0	n.a.	26.8 (21.5-35.9)	36.2 (20.8-43.2)	45.5 (26.8-50.0)
IL-10	T helper cells	6.1	9.8	25.8	4.2 (3.0-9.8)	5.8 (2.9-14.7)	22.0 (15.5-24.6)
	Cytotoxic T cells	11.6	17.0	25.7	5.1 (4.0-11.7)	10.3 (5.3-24.1)	24.3 (9.8-31.4)

	B cells	19.1	12.5	11.4	11.4 (9.6-16.5)	8.5 (4.6-14.5)	4.3 (3.8-5.4)
	Granulocytes	5.6	6.9	n.a.	0.3 (0.2-0.6)	0.6 (0.3-2.9)	n.a.
	Monocytes	62.8	43.7	n.a.	19.7 (16.5-22.0)	7.8 (1.3-5.6)	n.a.
CD25/ FoxP3	CD4+ cells	0.5	2.1	5.4	0.6 (0.2-1.0)	1.0 (0.4-1.9)	14.9 (4.2-45.1)
