# Supplementary material for: A novel direct co-culture assay analyzed by multicolor flow cytometry reveals context- and cell type-specific immunomodulatory effects of equine mesenchymal stromal cells
Source: PLoS One. 2019 Jun 27;14(6):e0218949. doi: 10.1371/journal.pone.0218949 (PMC6597077; doi:10.1371/journal.pone.0218949)
Supplement: S2 Table — (PDF) [file pone.0218949.s002.pdf]

S2 Table:

Primary antibodies, isotype controls and secondary antibodies

**Primary antibodies**							
**Antigen**	Conjugate	Host	Isotype	Clone	Reactivity	Supplier	Dilution
**CD3**	FITC	Rat	IgG1	CD3-12	Human	Bio-Rad AbD Serotec, Puchheim, Germany	1:50
**CD4**	FITC	Mouse	IgG1	CVS4	Horse	Bio-Rad AbD Serotec, Puchheim, Germany	1:100
**CD8**	PE unconjugated	Mouse	IgG2a	CVS21	Horse	Bio-Rad AbD Serotec, Puchheim, Germany	1:10
**CD14**	APC	Mouse	IgG1	134620	Human	R&D Systems, Wiesbaden, Germany	1:50
**CD25**	Biotinylated	Mouse	IgG1	Not available	Horse	Dr Bettina Wagner, Cornell University, Ithaca, NY, USA [40]	1:50
**FoxP3**	PerCP-Cy5.5	Rat	IgG2a	FJK-16s	Mouse/ rat	Thermo Fisher Scientific, Darmstadt, Germany	1:100
**IFN-γ**	Alexa Fluor 647	Mouse	IgG1	CC302	Bovine	Bio-Rad AbD Serotec, Puchheim, Germany	1:100
**IL-1**	unconjugated	Mouse	IgG1	608714	Horse	R&D Systems, Wiesbaden, Germany	1:100
**IL-10**	APC	Mouse	IgG1	CC320	Horse	Bio-Techne, Wiesbaden-Nordenstadt, Germany	1:400

**MAC**	unconjugated	Mouse	IgG1	MAC387	Human	Bio-Rad AbD Serotec, Puchheim, Germany	1:100
**TNF-α**	unconjugated	Mouse	IgG2b	CC327	Bovine	Bio-Rad AbD Serotec, Puchheim, Germany	1:50

Isotype controls (IC)

**Corresponding primary antibodies**	Conjugate	Host	Isotype	Clone	Supplier	Dilution
**Anti-CD3**	FITC	Rat	IgG1	Not available	Bio-Rad Laboratories, Munich, Germany	1:50
**Anti-CD4**	FITC	Mouse	IgG1	MOPC-21	BioLegend, Koblenz, Germany	1:100
**Anti-CD8**	Alexa 700	Mouse	IgG2a	MOPC-173	BioLegend, Koblenz, Germany	1:100
**Anti-CD14**<br>**Anti-IL-10**	APC	Mouse	IgG1	MOPC-21	BioLegend, Koblenz, Germany	1:50<br>1:400
**Anti-CD25**	biotinylated	Goat	Polyclonal IgG	Not available	R&D Systems, Wiesbaden, Germany	1:50
**Anti-FoxP3**	PerCP-Cy5.5	Rat	IgG2a	eBR2a	Thermo Fisher Scientific, Darmstadt, Germany	1:100
**Anti-IFN-γ**	Alexa Fluor 647	Mouse	IgG1	MOPC-21	BioLegend, Koblenz, Germany	1:100
**Anti-IL-1**	unconjugated	Mouse	IgG1	MG1-45	BioLegend, Koblenz, Germany	1:100

**Anti-MAC**	Alexa Fluor 700	Mouse	IgG1	11711	R&D Systems, Wiesbaden, Germany	1:100
**Secondary antibodies**						
**Target primary antibodies**	Conjugate		Host	Reactivity	Supplier	Dilution
**Anti-CD8**<br>**Anti-MAC**	Alexa Fluor 700		Goat IgG (H+L)	Mouse	Thermo Fisher Scientific, Damstadt, Germany	1:200
**Anti-CD25**<br>**IC biotinylated goat IgG**	BV605		none	Streptavidin	BioLegend, Koblenz, Germany	1:50
**Anti-IL-1**<br>**Isotype control mouse IgG1**	Texas Red		Goat IgG	Mouse	Bio-Rad AbD Serotec, Puchheim, Germany	1:500
**Anti-TNF-α**<br>**IC rabbit IgG**	PE		Rabbit F(ab’)2 IgG	Mouse	Bio-Rad AbD Serotec, Puchheim, Germany	1:50
