# Supplementary figures and images for: A novel direct co-culture assay analyzed by multicolor flow cytometry reveals context- and cell type-specific immunomodulatory effects of equine mesenchymal stromal cells
Source: PLoS One. 2019 Jun 27;14(6):e0218949. doi: 10.1371/journal.pone.0218949 (PMC6597077; doi:10.1371/journal.pone.0218949)

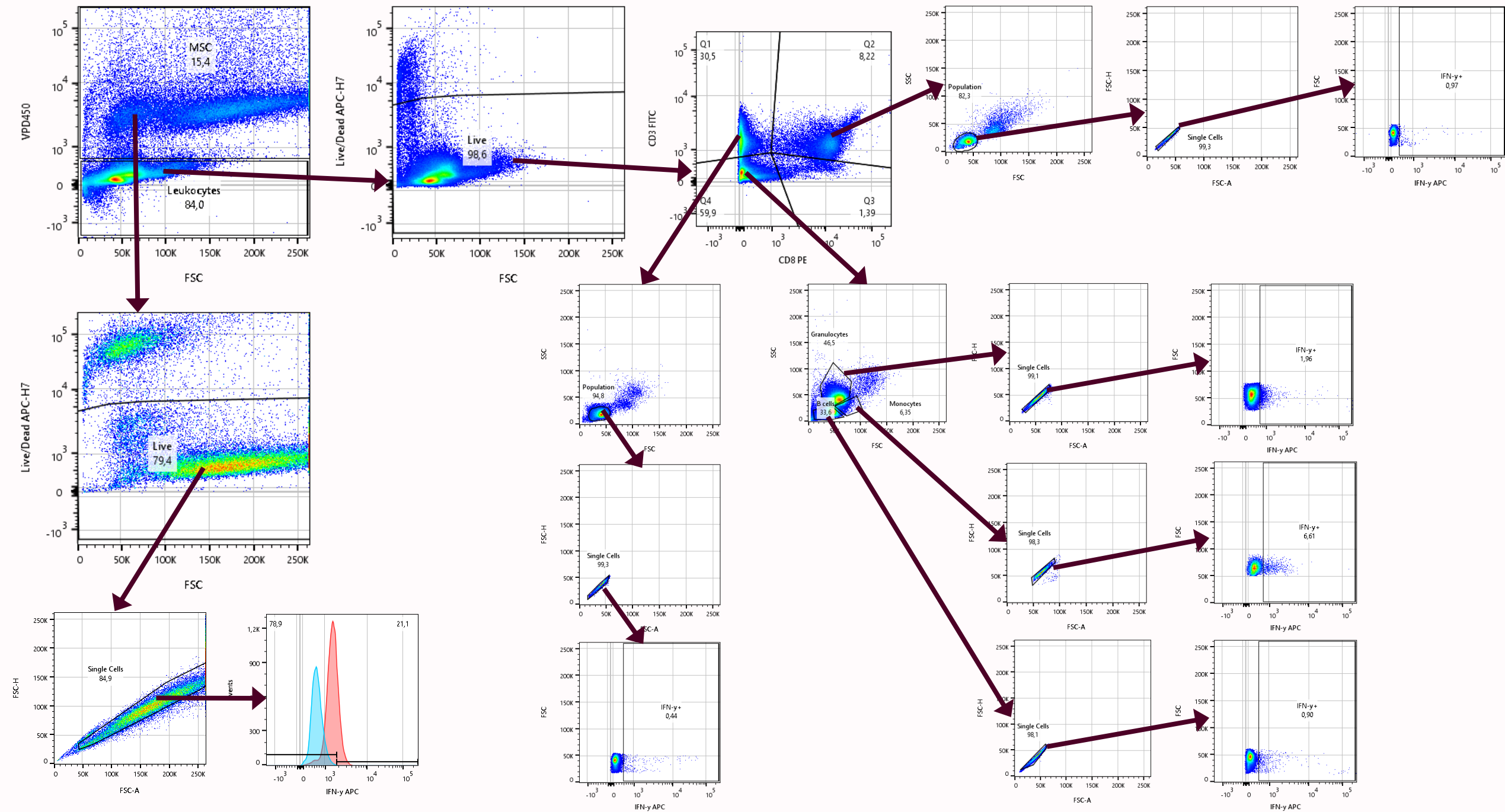

Supplement: S1 Fig — The figure shows systematic gating strategies based on FMO, isotype and Live/Dead controls for samples stained with CD3, CD8 and IFN-γ. A co-cultured sample of MSC and ConA-activated leukocytes was used to create the figure. (PDF) [file pone.0218949.s003.pdf]

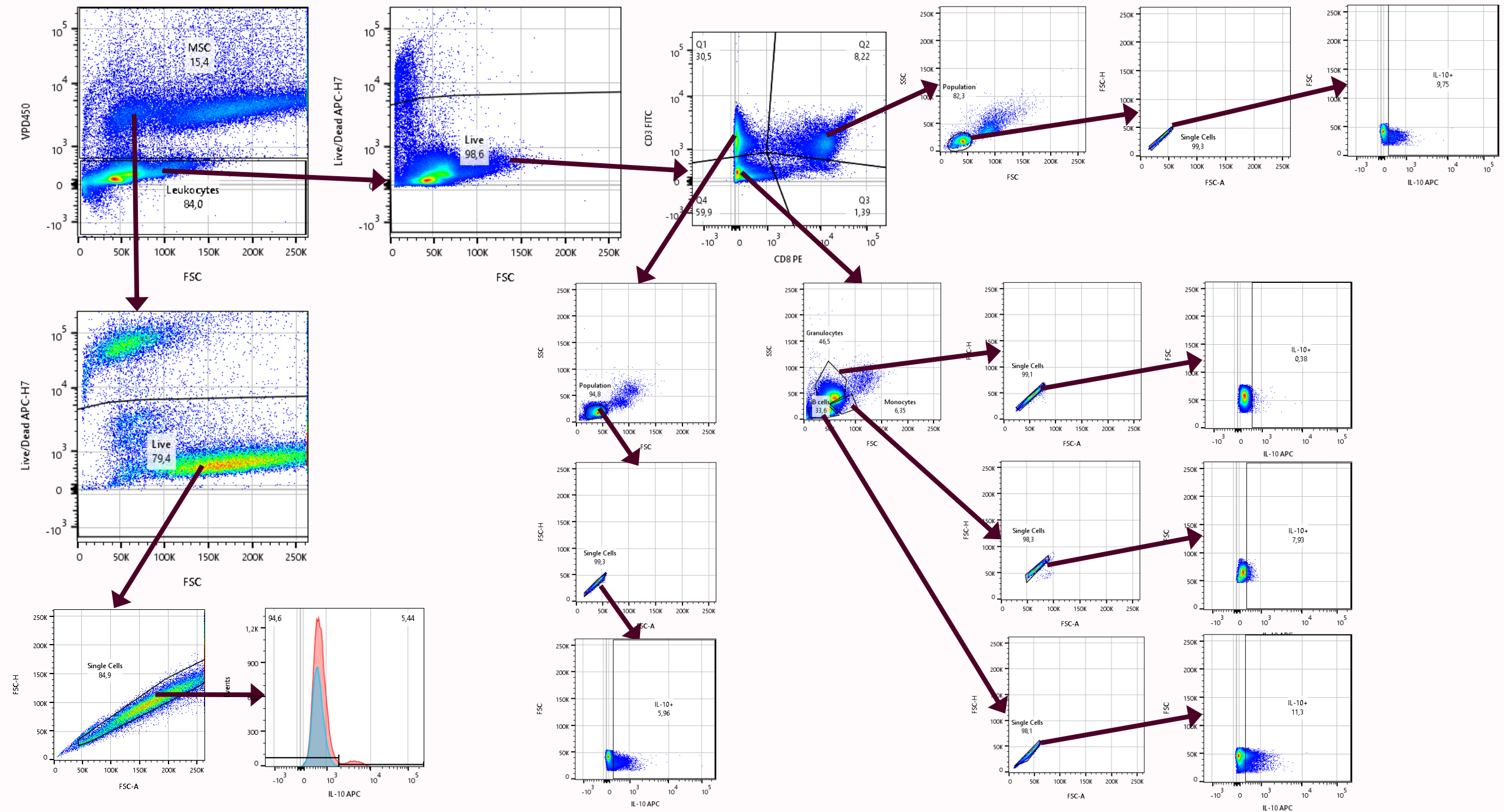

Supplement: S2 Fig — The figure shows systematic gating strategies based on FMO, isotype and Live/Dead controls for samples stained with CD3, CD8 and IL-10. A co-cultured sample of MSC and ConA-activated leukocytes was used to create the figure. (PDF) [file pone.0218949.s004.pdf]

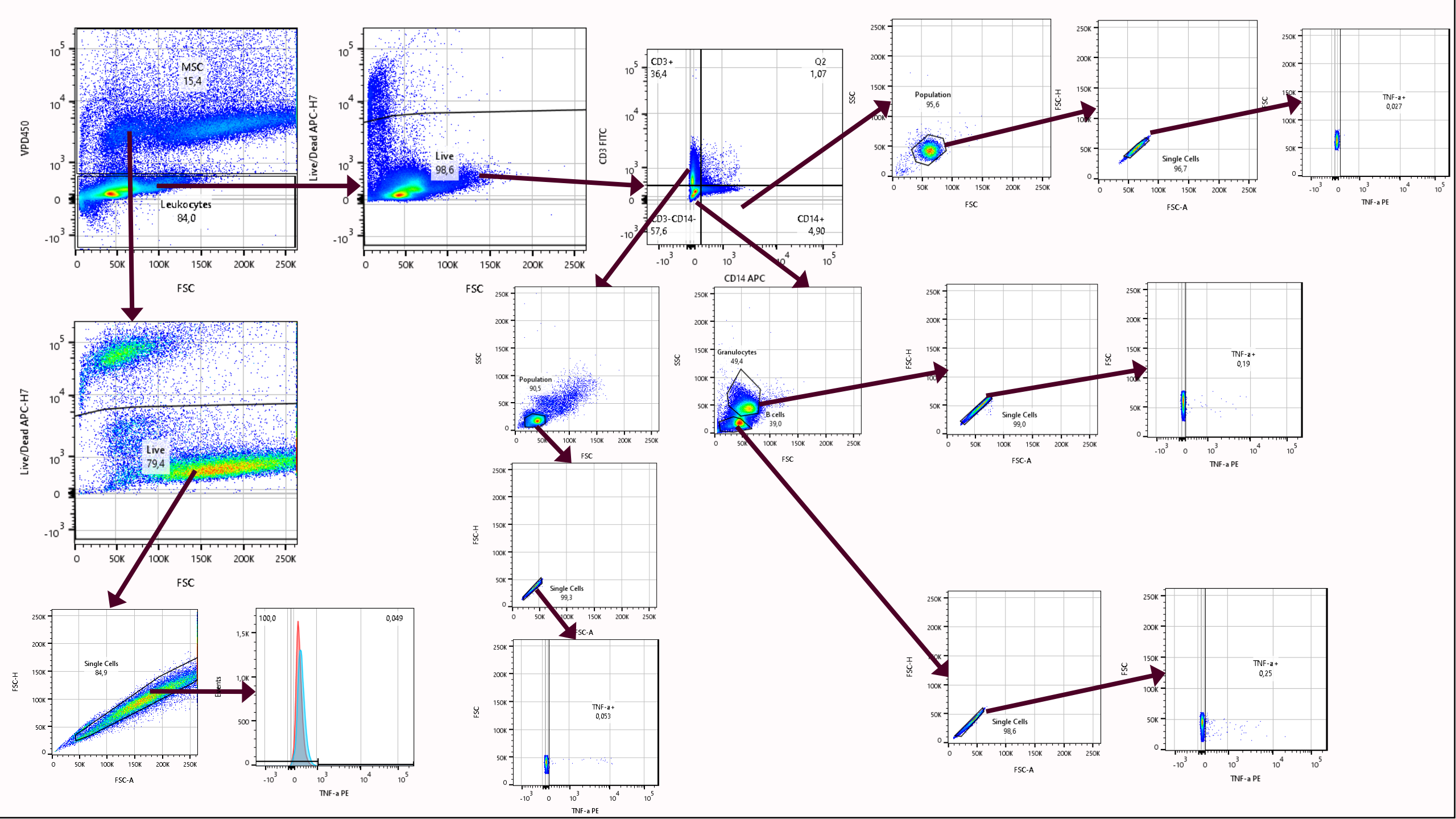

Supplement: S3 Fig — The figure shows systematic gating strategies based on FMO, isotype and Live/Dead controls for samples stained with CD3, CD14 and TNF-α. A co-cultured sample of MSC and ConA-activated leukocytes was used to create the figure. (PDF) [file pone.0218949.s005.pdf]

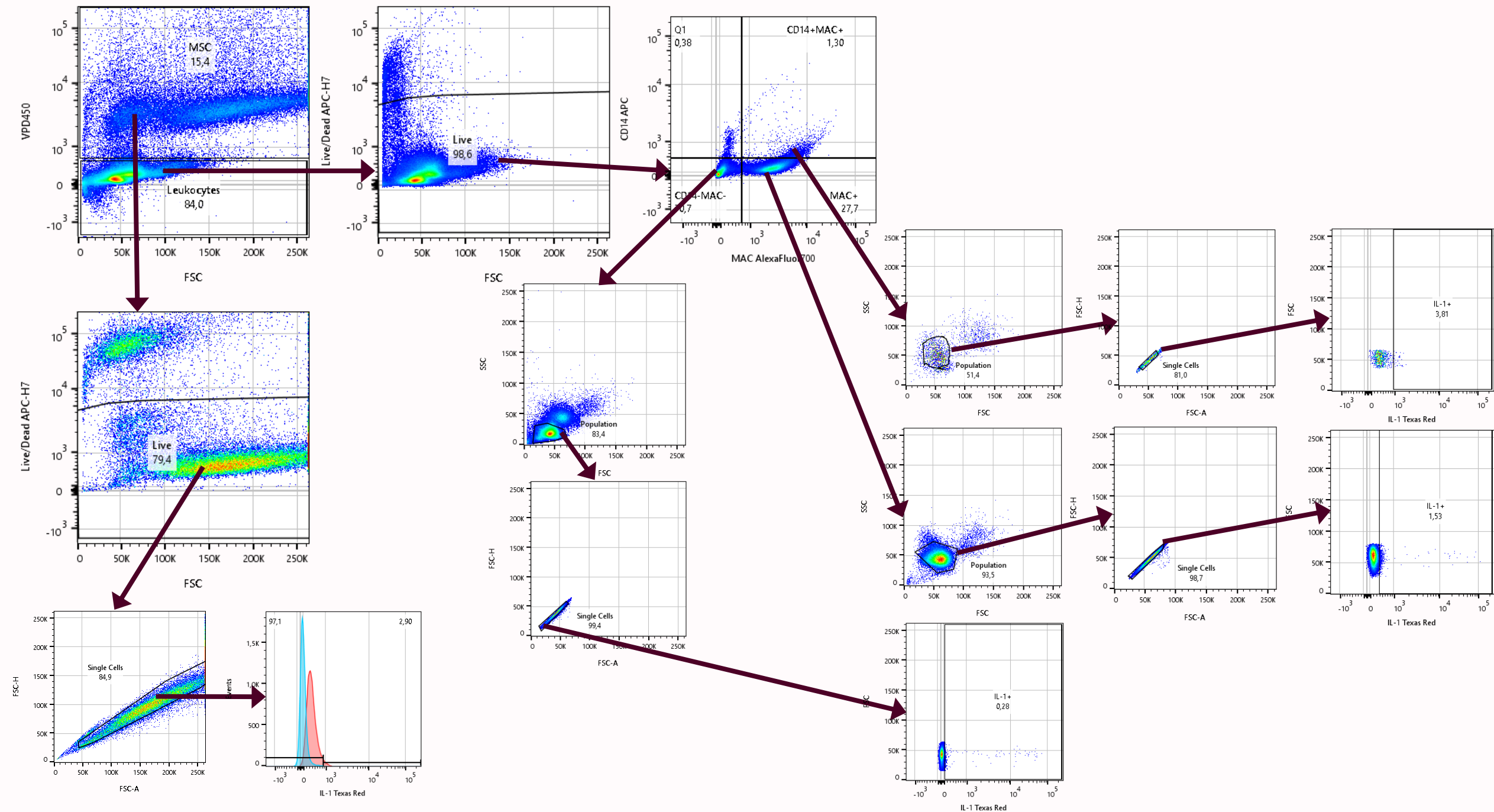

Supplement: S4 Fig — The figure shows systematic gating strategies based on FMO, isotype and Live/Dead controls for samples stained with CD14, MAC and IL-1. A co-cultured sample of MSC and ConA-activated leukocytes was used to create the figure. (PDF) [file pone.0218949.s006.pdf]

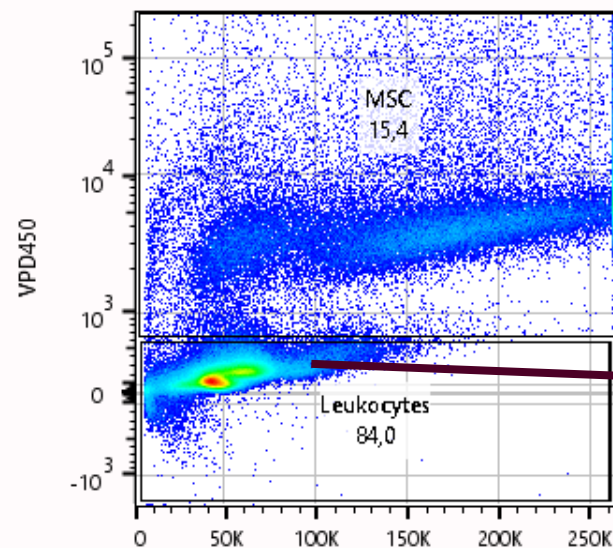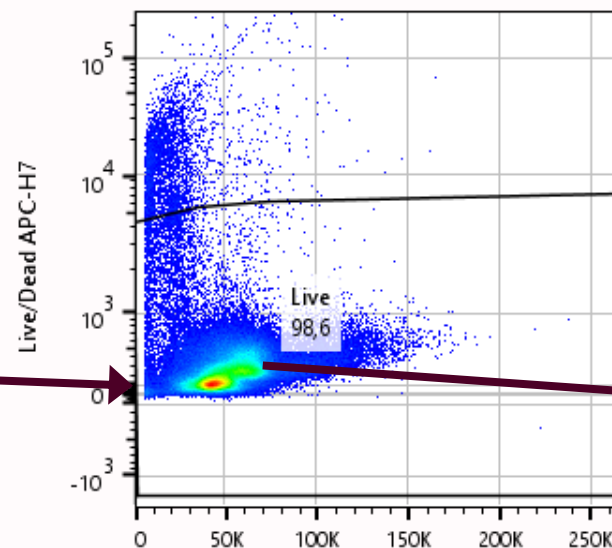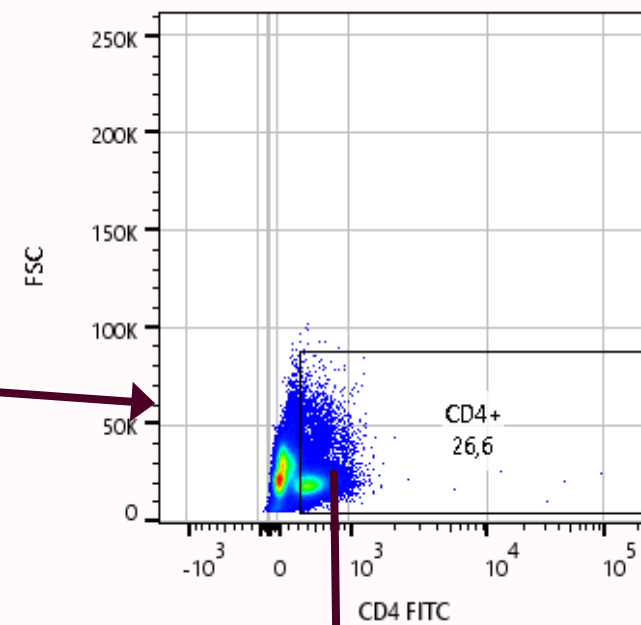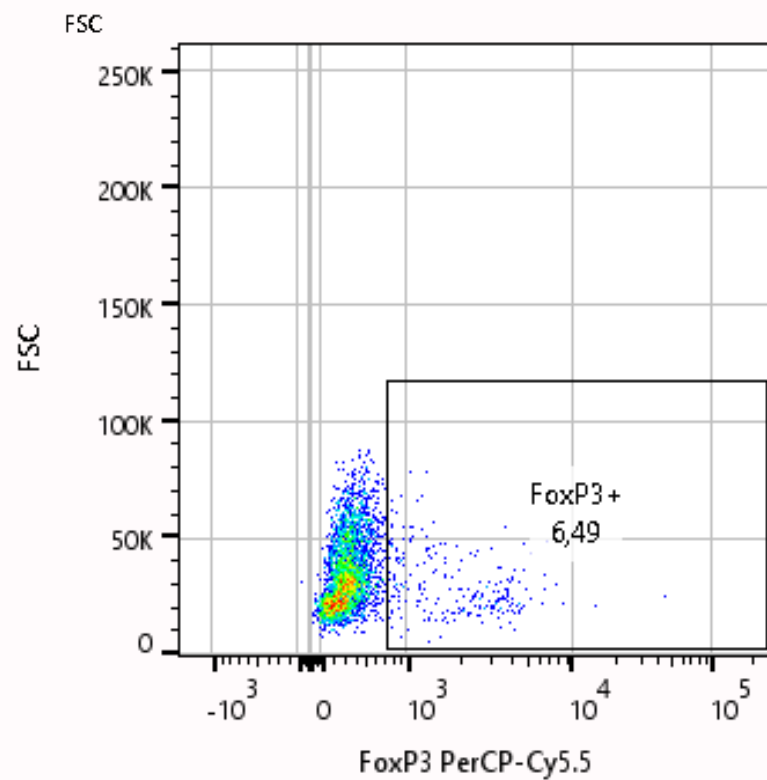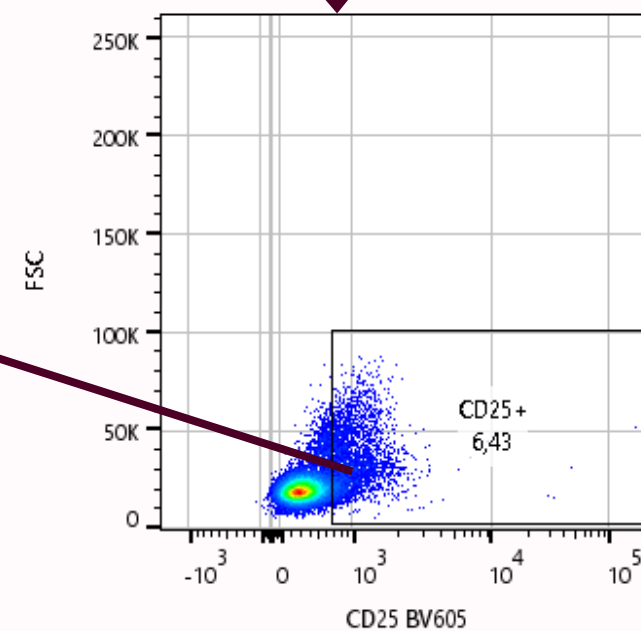

Supplement: S5 Fig — The figure shows systematic gating strategies based on FMO, isotype and Live/Dead controls for samples stained with CD4, CD25 and FoxP3. A co-cultured sample of MSC and ConA-activated leukocytes was used to create the figure. (PDF) [file pone.0218949.s007.pdf]
